# No acceleration of recovery from exercise-induced muscle damage after cold or hot water immersion in women: A randomised controlled trial

**DOI:** 10.1371/journal.pone.0322416

**Published:** 2025-05-07

**Authors:** Vanessa Wellauer, Ron Clijsen, Giannina Bianchi, Emilia Riggi, Erich Hohenauer

**Affiliations:** 1 Rehabilitation and Exercise Science Laboratory (RESlab), Department of Business Economics, Health and Social Care, University of Applied Sciences and Arts of Southern Switzerland, Landquart, Switzerland; 2 International University of Applied Sciences THIM, Landquart, Switzerland; 3 Department of Movement and Sport Sciences, Vrije Universiteit Brussel, Brussels, Belgium; 4 Health Department, Bern University of Applied Science, Bern, Switzerland; 5 Department of Business Economics, Health and Social Care, University of Applied Sciences and Arts of Southern Switzerland, Manno, Switzerland; 6 Department of Neurosciences and Movement Science, University of Fribourg, Fribourg, Switzerland; University of Hertfordshire, UNITED KINGDOM OF GREAT BRITAIN AND NORTHERN IRELAND

## Abstract

This study compared the effects of cold water immersion (CWI) and hot water immersion (HWI) on muscle recovery following a muscle-damaging exercise protocol in women. Thirty healthy women (23.3 ± 2.9 years) were randomly assigned to either the CWI, HWI, or control (CON) groups. Participants completed a standardised exercise protocol (5 x 20 drop-jumps), followed by a 10 min recovery intervention (CWI, HWI, or CON) immediately and 120 min post-exercise. Physiological responses, including muscle oxygen saturation (SmO_2_), core and skin temperature, and heart rate, were assessed at baseline, immediately post-exercise, after the first recovery intervention (postInt), and during 30 min follow-up. Recovery was evaluated through maximal voluntary isometric contraction, muscle swelling, muscle soreness ratings, and serum creatine kinase at baseline, 24, 48, and 72 h post-exercise. A mixed-effects model was used to account for repeated measures over time. Results showed lower SmO_2_ values in the CWI compared to the HWI group at 20 min (Δ-6.76%, CI: −0.27 to −13.25, p = 0.038) and 30 min (Δ-9.86%, CI: −3.37 to −16.35, p = 0.001), and compared to CON at 30 min (Δ-7.28%, CI: −13.77 to −0.79, p = 0.022). Core temperature was significantly higher in the HWI than the CWI group (postInt and 30 min), while it was significantly lower in the CWI group than CON (30 min). CWI caused a substantial decrease in skin temperature compared to HWI and CON between postInt and 30 min follow-up (all p < 0.001). Skin temperature was higher in the HWI group compared to CON at postInt and throughout 30 min follow-up (all p < 0.001). No significant differences in recovery markers were observed between CWI and HWI groups, although HWI led to slightly higher creatine kinase levels (24 h and 72 h) and greater muscle swelling (24 h) compared to CON. Despite distinct acute physiological responses to CWI and HWI, neither improved subjective or objective recovery outcomes during the 72 h follow-up compared to CON in women following a muscle-damaging exercise protocol.

**Trial registration number:**

NCT04902924 (ClinicalTrials.gov), SNCTP000004468 (Swiss National Clinical Trial Portal).

## Introduction

Unaccustomed physical exercise can lead to exercise-induced muscle damage (EIMD). The physiological response varies individually and mainly depends on the type of contraction, genetic factors, muscle group being trained, exercise volume, intensity, and type of exercise [1]. Mechanical stress can cause microscopic damage to muscle tissue, characterised by an inflammatory response in the days following exercise. Recovery from muscle damage is typically assessed using subjective and objective markers of recovery (e.g., loss of strength, delayed onset of muscle soreness [DOMS], upregulation of pro-inflammatory muscle proteins, such as creatine kinase [CK], and oedema-induced muscle swelling) [2,3]. While the inflammatory response is fundamental to tissue repair processes [4], excessive symptoms of EIMD can lead to prolonged recovery times with impaired physical performance. Various post-exercise interventions, including water immersion, cold air exposure, massage, and active or passive recovery, have emerged as potential techniques to accelerate recovery [5,6].

Of particular interest is post-exercise water immersion, which is a commonly used recovery strategy [7,8] and is of relatively low cost. The hydrostatic pressure during immersion can cause a shift of fluids from the extremities towards the central cavity, leading to increased cardiac output, promoting translocation of metabolites from the muscles, accelerating substrate transport and reducing oedema [9]. In addition, cold water immersion (CWI) decreases superficial and subcutaneous tissue temperature, inducing local vasoconstriction in the immersed body parts. A reduction in cellular metabolism is reported to be the primary mechanism for reducing the acute inflammatory response and, consequently, muscle swelling [9,10]. On the other hand, hot water immersion (HWI) increases subcutaneous and cutaneous tissue temperatures, causing peripheral vasodilation and an increase in blood flow of the affected areas, metabolism, nutrient delivery and removal of waste by-products [9].

Several studies have investigated the influence of post-exercise CWI on acute physiological changes or recovery (24–72 h) [11–14]. Its effectiveness appears to depend strongly on the type of the previous exercise performed [8,15,16]. However, unlike CWI, there is limited data on HWI used as a recovery intervention [15], with mixed results regarding its effectiveness. While a recently published study found no beneficial effect of post-resistance exercise HWI compared to a control group (CON) on recovery (muscle function, muscle soreness, and blood markers) [17], others reported advantages of HWI compared to CON in recovery of strength [18,19], lower creatine kinase activity and ratings of muscle soreness [19]. In addition, recent evidence by Sautillet et al. suggests maintaining a core body temperature between 38.5°C and 39°C during HWI may significantly improve muscle recovery in active males [20]. Recent reviews summarise that CWI has generally been shown to be a more effective recovery method following strenuous exercise than other modalities- such as active recovery, contrast water therapy, warm water immersion, air cryotherapy, and massage- for reducing DOMS and pro-inflammatory CK levels. However, directly comparing the efficacy of post-exercise CWI and HWI on recovery is challenging due to the variability in protocols, making it nearly impossible to draw definitive conclusions and make practical recommendations.

To date, literature directly comparing the effects of post-exercise CWI, and HWI on recovery is scarce. To our knowledge, existing studies have only been conducted on male subjects. Female participants are significantly underrepresented in sports and exercise medicine research, including studies related to CWI and HWI [7,21]. Hormonal status (e.g., estrogen levels) and differences in body composition, such as the amount and distribution of subcutaneous adipose tissue [22], could potentially contribute to divergent outcomes following muscle-damaging exercise regimens and water immersion in women. Therefore, this study aimed to compare the physiological responses and effects of post-exercise CWI and HWI on subjective and objective recovery characteristics in a female population. We hypothesised that CWI could have a more significant impact on the physiological response and, therefore, would be a more effective method than HWI for enhancing subjective recovery (DOMS) and objective recovery, measured as MVIC recovery.

## Methods

### Study design

**Fig 1:**
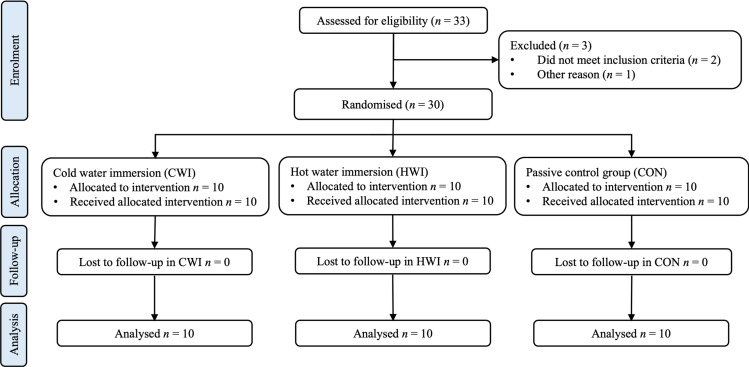
CONSORT flowchart of participants.

The trial followed a parallel-group study design. Participants were randomly assigned to one of three intervention groups (CWI, HWI, and CON) using block randomisation with an allocation ratio of 1:1:1. A block of 30 participants was used to ensure an equal distribution across the groups. Randomisation was done using 30 opaque envelopes, each containing a number (1, 2 or 3). The investigator (EH) prepared all envelopes and made them available to the participants for random selection. Participants were assigned to their respective groups based on the number drawn from the envelope (Fig 1). Due to the nature of the study, blinding of participants and assessors to the recovery intervention was not feasible. Based on previous studies with a similar methodological design [23,24], an *A priori* sample size calculation for repeated-measures ANOVA, within-between interaction (G*Power, V 3.1, Franz Faul, Germany) with an estimated effect size of 0.3, alpha = 0.05 and power of 0.8 was conducted. According to these specifications, a sample size of n = 24 was estimated. To account for a potential loss of data of 20% owing to dropouts and incomplete records, the target sample size was set at 30. There were no dropouts among the selected participants. The study was registered at the clinialtrials.gov registry (NCT04902924) and approved by the local Ethics Committee of Zurich (2021–00546) in accordance with the Declaration of Helsinki (ICH-GCP). All measurements were performed between October 2021 and April 2022 in the Laboratory of the University of Applied Sciences and Arts of Southern Switzerland (RESlab, Landquart, Switzerland).

### Participants

A total of n = 30 recreationally active volunteers were included. Women aged between 18 and 35 years old with no previous surgery on the musculoskeletal system of the trunk and lower limbs were eligible for the study. Participants were excluded if they were smokers, pregnant, had acute injuries or pain, had injuries in the trunk area or lower extremities within less than one year, or were taking medication. All participants were fully informed of the aims, risks, and discomforts related to the experimental protocol of the study and provided written informed consent before participating. The characteristics of the study participants are presented in Table 1.

**Table 1:** Demographic data of female participants (n = 30).

Parameters	CWI (n = 10)	HWI (n = 10)	CON (n = 10)
Body height [cm]	166.9 ± 7.8	171.0 ± 5.5	165.0 ± 6.8
Body weight [kg]	61.3 ± 8.7	68.7 ± 9.2	58.8 ± 6.7
Lower body fat mass [%]	31.6 ± 2.9	33.9 ± 4.2	29.3 ± 3.9
Age [years]	23.8 ± 3.3	23.1 ± 3.6	23.1 ± 1.6
BSA [m^2^]	1.69 ± 0.15	1.80 ± 0.13	1.64 ± 0.11
BSA/mass [m^2^/kg]	0.03 ± 0.001	0.03 ± 0.002	0.03 ± 0.002
BMI [kg/m^2^]	21.9 ± 2.0	23.4 ± 2.4	21.6 ± 2.2
Menstruation ph. [%]	30	0	10
Proliferative ph. [%]	50	40	40
Secretory ph. [%]	20	60	50

BSA = body surface area, BMI = body mass index, ph = phase; values are mean ± SD.

### Experimental overview

**Fig 2:**
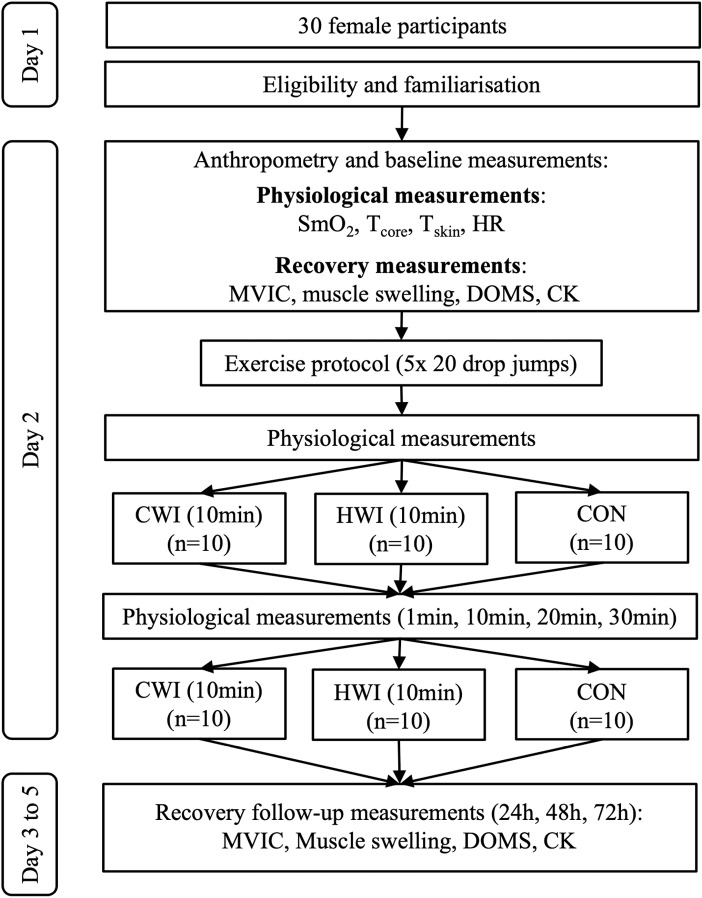
Schematic representation of the experimental protocol over time. CWI cold water immersion, HWI hot water immersion, CON control, SmO_2_ muscle oxygen saturation, T_skin_ skin temperature, T_core_ core temperature, HR heart rate, MVIC maximum voluntary isometric contraction, DOMS delayed onset of muscle soreness, CK creatine kinase.

Experiments were completed over five days. On day 1, participants completed a screening questionnaire to determine eligibility and subsequently signed the informed consent form. Subjects were instructed to refrain from alcohol, dietary supplements, or additional exercise during the experimental period. They were familiarised with the experimental setup of the maximum voluntary isometric contraction (MVIC) test on a custom-designed ergometer chair. One week after familiarisation, on experimental day 2, standing body height was assessed using a GPM stadiometer (Zurich, Switzerland). The TANITA-TBF 611 scale (Tokyo, Japan) was used to measure body weight and estimate lower body fat percentage. Body mass index (BMI) [25], body surface area (BSA) [26], and BSA to mass ratio were calculated from the height and weight of participants. Anthropometry, menstrual cycle (using the calendar counting method), and baseline data were recorded before performing the exercise protocol. Immediately after exercise and two hours later, participants underwent their assigned recovery interventions. All physiological parameters were measured at baseline (BL), directly following exercise (postEx), and 1 min (postInt), 10 min, 20 min, and 30 min after the first recovery intervention. Markers of muscle recovery were evaluated at BL, 24 h (experimental day 3), 48 h (day 4), and 72 h (day 5) after exercise. Experiments were conducted without any deviation from the protocol. A schematic representation of the experimental protocol is illustrated in Fig 2.

### Muscle-damaging exercise protocol

A validated, muscle-damaging exercise protocol (5 x 20 drop-jumps from a 0.6 m box), previously described in the literature [23], was employed to induce damage to the knee extensor muscles [2,24,27]. The participants were verbally encouraged, and the correct execution of the protocol was visually monitored. Participants were not allowed to drink water between BL and the 30 min follow-up period due to the potential influence of ingested water on core temperature (T_core_) measurements.

### Recovery modalities

Participants in the CWI and HWI groups were immersed up to the level of the sternum in a square inflatable pool (length: 168 cm, width: 168 cm, height: 70 cm) filled with cold (10 ± 0.5°C) and hot (40 ± 0.5°C) water respectively. The water temperature and duration of 10 min were set as described previously [24,28–30]. Water temperature was controlled with a thermometer (Voltcraft MT52 digital multimeter, Hirschau, Germany) and ice or hot water was added if necessary. Two hours after the first CWI or HWI, a second set of immersions of identical duration (10 min) and water temperature (10°C or 40°C) was performed. CON received no treatment and recovered for 10 minutes in a supine position in a room with an ambient temperature of 21 ± 2°C and a relative humidity of 40 ± 5%.

### Physiological outcomes

#### Muscle oxygen saturation.

To quantify muscle oxygen saturation (SmO_2_) of the knee extensor muscles, a deep tissue oxygenation monitor (moorVMS-NIRS, Moor Instruments, Millwey, UK) was used. The detector consisting of two identical photodiodes and the emitter were placed 30 mm from each other on the muscle belly of the right quadriceps femoris muscle secured with adhesive tape (Hypafix, BSN, Hamburg, Germany), as described previously [31]. Oxygenated haemoglobin (oxyHb), deoxygenated haemoglobin (deoxyHb) and total haemoglobin (TotHb) were assessed in arbitrary units and SmO_2_ was calculated as oxyHb/TotHb x 100 value (%). Near-infrared spectroscopy (NIRS) provides a non-invasive valid and reliable assessment tool for skeletal muscle oxidative metabolism [32].

#### Heart rate.

HR was recorded using a Polar watch (Vantage M2, Polar, Kempele, Finland) and a Bluetooth chest belt (H10, Polar, Kempele, Finland) in beats per minute (bpm).

#### Core temperature.

T_core_ was monitored continuously using the gastrointestinal telemetry capsule e-Celsius and receiver e-Viewer (Body Cap, Caen, France). Participants were required to ingest the e-Celsius capsule upon entry into the laboratory (day 2). Data was recorded at 30s intervals and transmitted for further analysis. E-Celsius is a valid system for measuring T_core_ with good test-retest reliability [33,34], which tends to underestimate rectal temperature by ~0.2°C during exercise [33,35] and rest [35].

#### Skin temperature.

T_skin_ of the right thigh was evaluated using infrared thermal imaging (FLIR A600 series, Emitec Industrial, Rotkreuz, Switzerland) and the corresponding data analysis software (FLIR ResearchIR Max). The infrared camera’s emissivity factor was adjusted to 0.954. In a predefined skin area on the midsection of the right frontal thigh, the region of interest (covering 3650 px) was manually marked in the software. The average temperature of this area was utilised for analysis.

### Recovery outcomes

#### Muscle soreness.

DOMS of the knee extensor muscles was evaluated subjectively using a 10 cm Visual Analogue Scale (VAS) during isometric squatting at a 90° knee angle after 3s [36]. The VAS ranged from 0 cm, indicating “no soreness”, to 10 cm, indicating “severe soreness”. DOMS is considered a practical and indirect marker of muscle damage [37].

#### Muscle strength.

Muscle strength of the right knee extensor was assessed based on the performance of a MVIC on a custom-designed ergometer chair. Participants sat upright on the chair with the knee flexed at 120°, hip angle of 100°, and the right shin fixed to the ergometer with a strap to ensure isometric contraction [12]. For evaluation of muscle strength, participants maximally extended their knees three times for 4s, with each session separated by a 2-min break. No verbal encouragement was given, and the subjects were blinded to the MVIC values. The highest value from the three attempts was used as the MVIC (in N) for the analysis.

#### Muscle swelling.

Oedema-induced muscle swelling of the right knee extensor was assessed based on acute changes in muscle thickness measured via ultrasound (MyLabClass C, Esaote, Genoa, Italy) using B-mode. Participants lay in a supine position and a minimal pressure technique was applied. The area of interest was defined as 60% of the distance between the greater trochanter and lateral epicondyle, 3 cm lateral to the midline [23,24,38] and labelled with a waterproof marker to ensure reliability throughout the experimental period. Muscle thickness was determined as the distance (in mm) between the femoral bone and the outer layer of the quadriceps muscle (excluding the overlying adipose tissue). The mean distance of three images was calculated (Pixmeo SARL, OsiriX V.8.0.2., Bernex, Switzerland). Ultrasound has been demonstrated to be a valid and reliable tool to measure muscle thickness [39].

#### Concentration of serum CK.

Blood samples were obtained from the antecubital fossa to assess serum CK concentration (%). Samples were collected in 8.5mL tubes (BD Vacutainer, Plymouth, UK), centrifuged at 3000g for 10 min (Hettich, EBA 20, Baech, Switzerland) and analysed using an automated ultraviolet method (Roche, Basel, Switzerland). CK is an indirect blood marker of muscle damage commonly used to indicate muscle membrane permeability. However, given the significant inter- and intra-subject variability of CK, other indirect markers, such as loss of muscle strength and DOMS, should also be considered for a more accurate assessment of muscle damage [2,40].

### Statistical analysis

Details of the demographics and anthropometric characteristics of participants are presented in Table 1. For each outcome variable, physiological and recovery parameters, a mixed-effects model with a restricted maximum likelihood method was fitted to account for repeated measures over time. Subjects were included as a random effect, while the intervention (CWI, HWI, CON), time points (for physiological parameters: BL, postEx, postInt, 10, 20, 30 min; for recovery parameters: BL, 24, 48, 72 h) and their interaction were included as fixed effects. All these models were adjusted for body fat (%) and the baseline level of the outcome being analysed. As the estimated lower body fat was the only baseline characteristic that differed significantly between the three intervention groups, it was included as an adjustment in the mixed-effect models. After fitting the model, pairwise comparisons of the intervention effects (CWI vs CON, HWI vs. CON, HWI vs CWI) were tested both overall and at each time point using the Bonferroni correction for multiple comparisons. To measure the magnitude of the difference between groups, we also reported the Hedge’s G effect size as supplementary material (Table S3 and S4). The menstrual cycle was assessed as descriptive data and was not included as a covariate in the mixed-effects model. Statistical analyses were performed using Stata V18 (StataCorp LLC, Texas, USA), with the significance level set at p < 0.05. Figures were created using Prism 9 (GraphPad, Software Inc.).

## Results

### Physiological parameters

#### Muscle oxygenation saturation.

**Fig 3:**
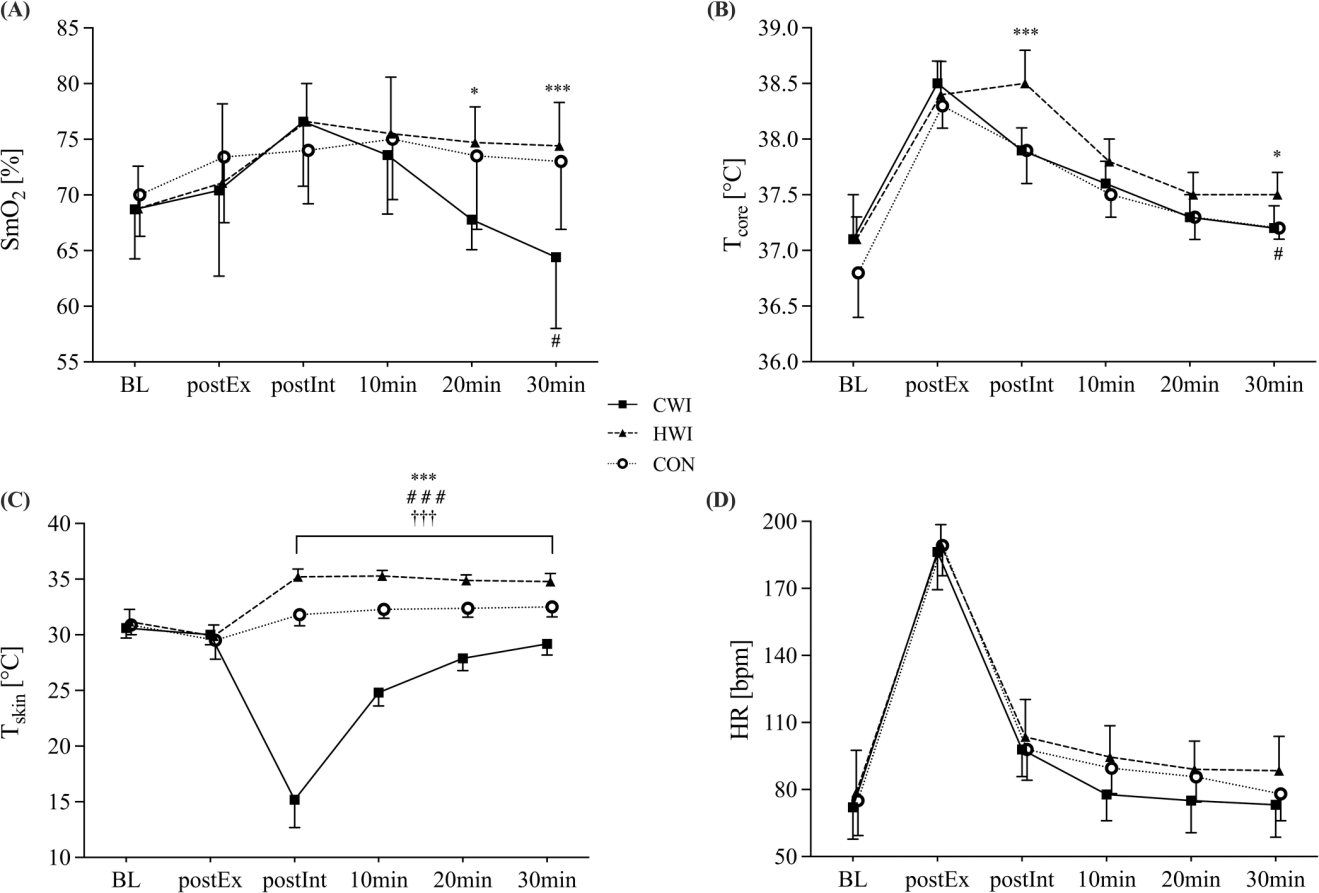
Results of physiological parameters. (A) muscle oxygen saturation of the right quadriceps femoris muscle (SmO_2_), (B) core temperature (T_core_), (C) skin temperature (T_skin_), and (D) heart rate (HR) over time in CWI, HWI and CON groups. All data are presented as mean±SD. BL baseline, postEx post-exercise, postInt post-intervention, ***p < 0.001 CWI vs HWI, *p < 0.05 CWI vs HWI, ^###^p < 0.001 CWI vs CON, ^#^p < 0.05 CWI vs CON, ^†††^p < 0.01 HWI vs CON.

Analysis of SmO_2_, using a joint test, revealed a significant main effect of time (p < 0.001) and a time*intervention interaction (p < 0.001), but no significant intervention effect (p = 0.105). Bonferroni-adjusted p-values indicated significant differences in between-group pairwise comparison at 20- and 30 min follow-up (Table 2, Fig 3A).

**Table 2:** Pairwise comparisons of the intervention effects by time point for physiological parameters.

Comparison	Muscle oxygen saturation [%]		Core temperature [°C]		Skin temperature [°C]		Heart rate [bpm]	
	β* (95% CI)	*P*-value	β* (95% CI)	*P*-value	β* (95% CI)	*P*-value	β* (95% CI)	*P*-value
**postEx**								
CWI vs CON	−1.68 (−8.17; 4.81)	1.000	−0.13 (−0.46; 0.19)	0.997	0.89 (−0.25; 2.03)	0.186	−0.00 (−11.74; 11.74)	1.000
HWI vs CON	−1.12 (−7.61; 5.37)	1.000	−0.16 (−0.49; 0.16)	0.685	0.15 (−0.99; 1.29)	1.000	−3.40 (−15.14; 8.34)	1.000
HWI vs CWI	0.56 (5.93; 7.05)	1.000	−0.03 (−0.36; 0.29)	1.000	−0.74 (−1.88; 0.40)	0.362	−3.40 (−15.14; 8.34)	1.000
**postInt**								
CWI vs CON	3.92 (−2.57; 10.41)	0.444	−0.27 (−0.59; 0.06)	0.152	−16.29 (−17.43; −15.15)	**<0.001**	2.90 (−8.84; 14.64)	1.000
HWI vs CON	3.78 (−2.71; 10.27)	0.489	0.24 (−0.08; 0.57)	0.226	3.10 (1.96; 4.24)	**<0.001**	2.00 (−9.74; 13.74)	1.000
HWI vs CWI	−0.14 (−6.63; 6.35)	1.000	0.51 (0.18; 0.83)	**0.001**	19.39 (18.25; 20.53)	**<0.001**	−0.90 (−12.64; 10.84)	1.000
**10 min**								
CWI vs CON	−0.08 (−6.57, 6.41)	1.000	−0.25 (−0.57; 0.08)	0.212	−7.14 (−8.28; −6.00)	**<0.001**	−8.80 (−20.54; 2.94)	0.218
HWI vs CON	1.68 (−4.81; 8.17)	1.000	−0.03 (−0.35; 0.30)	1.000	2.68 (1.54; 3.82)	**<0.001**	1.40 (−10.34; 13.14)	1.000
HWI vs CWI	1.76 (−4.73; 8.25)	1.000	0.22 (−0.11; 0.55)	0.318	9.82 (8.68; 10.96)	**<0.001**	10.20 (−1.54; 21.94)	0.113
**20 min**								
CWI vs CON	−4.38 (−10.87; 2.11)	0.318	−0.28 (−0.60; 0.05)	0.128	−4.21 (−5.35; −3.07)	**<0.001**	−7.70 (−19.44; 4.04)	0.349
HWI vs CON	2.38 (−4.11; 8.87)	1.000	−0.07 (−0.40; 0.25)	1.000	2.12 (0.98; 3.26)	**<0.001**	−0.30 (−12.04; 11.44)	1.000
HWI vs CWI	6.76 (0.27;13.25)	**0.038**	0.20 (−0.12; 0.53)	0.413	6.33 (5.19; 7.47)	**<0.001**	7.40 (−4.34; 19.14)	0.394
**30 min**								
CWI vs CON	−7.28 (−13.77; −0.79)	**0.022**	−0.36 (−0.69; −0.04)	**0.023**	−2.94 (−4.08; −1.80)	**<0.001**	−1.90 (−13.64; 9.84)	1.000
HWI vs CON	2.58 (−3.91; 9.07)	1.000	−0.02 (−0.35; 0.30)	1.000	1.95 (0.81; 3.09)	**<0.001**	6.70 (−5.04; 18.44)	0.516
HWI vs CWI	9.86 (3.37; 16.35)	**0.001**	0.34 (0.02; 0.67)	**0.037**	4.89 (3.75; 6.03)	**<0.001**	8.60 (−3.14; 20.34)	0.238

* Baseline time point as a reference. CWI = cold water immersion group, HWI = hot water immersion group, CON = control group, postEx = post-exercise, postInt = post-intervention. P-values adjusted for multiple comparisons with Bonferroni method.

#### Core temperature.

Significant main effects were observed for time (p < 0.001), intervention (p = 0.009), and time*intervention interaction (p = 0.002). Pairwise comparison showed significant between-group differences for T_core_ at postInt between the CWI and the HWI groups and at 30 min follow-up between the CWI and the HWI groups as well as between the CWI group and CON (Table 2, Fig 3B).

#### Skin temperature.

Evaluation of T_skin_ revealed significant main effects of time, intervention and a time*intervention interaction (all p < 0.001). T_skin_ was reduced in the CWI group compared to the HWI group and CON at postInt and throughout 30 min follow-up (Table 2; Fig 3C). In the HWI group, T_skin_ was significantly higher than CON at postInt and throughout the 30 min follow-up.

#### Heart rate.

For HR we found a significant main effect of time (p < 0.001) but not for intervention (p = 0.118) and time*intervention interaction (p = 0.051). Pairwise comparison showed no differences between the groups at any time point (Table 2, Fig 3D).

### Recovery parameters after the exercise task and intervention

#### Maximum voluntary isometric contraction.

**Fig 4:**
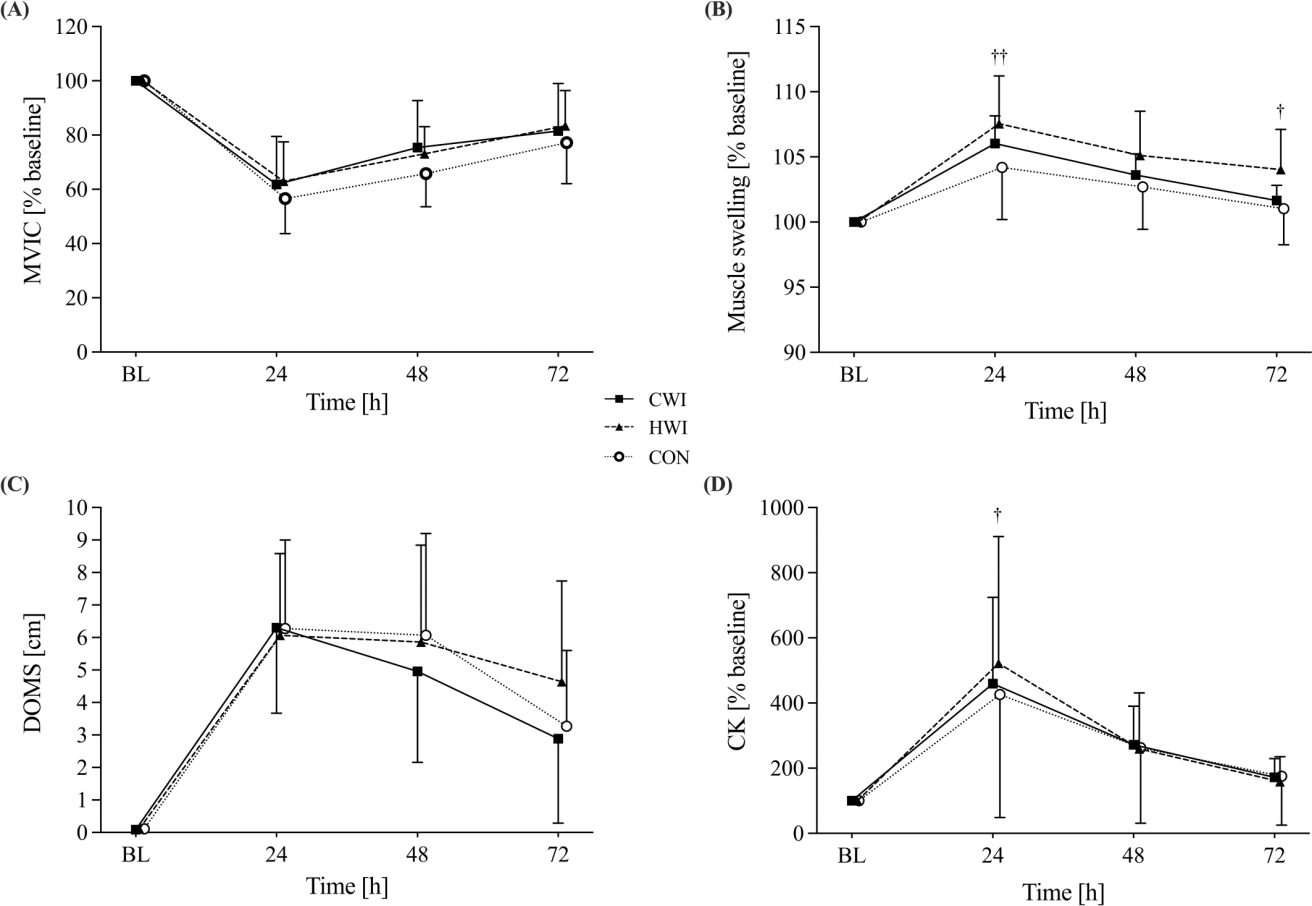
Results of recovery parameters. (A) maximum voluntary isometric contraction (MVIC), (B) muscle swelling, (C) delayed onset of muscle soreness (DOMS), and (D) creatine kinase (CK) over time in CWI, HWI and CON groups. A, B and D values are normalised to baseline (% mean±SD) for their initial values. All values are presented as mean±SD. BL baseline, ^††^p < 0.01 HWI vs CON, ^†^p < 0.05 HWI vs CON.

For MVIC, a significant time effect (p < 0.001) was detected, but no intervention (p = 0.573) and time*intervention interaction (p = 0.882) were observed (Fig 4A). No significant between-group differences were noted at any specific time point (Table 3).

**Table 3:** Pairwise comparisons of the intervention effects by time point for recovery parameter.

Comparison	Maximum voluntary isometric contraction [N]		Muscle swelling [mm]		Delayed onset of muscle damage [cm]		Concentration of CK [U/l]	
	β* (95% CI)	P-value	β* (95% CI)	P-value	β* (95% CI)	P-value	β* (95% CI)	P-value
**24 hours**								
CWI vs CON	1.90 (−7.61, 11.41)	1.000	0.07 (−0.06; 0.20)	0.607	0.04 (−2.54; 2.64)	1.000	127.30 (−140.12; 394.72)	0.763
HWI vs CON	−1.81 (−11.32; 7.70)	1.000	0.17 (0.04; 0.30)	**0.006**	−0.21 (−2.79; 2.37)	1.000	271.10 (3.68; 538.52)	**0.046**
HWI vs CWI	−3.71 (−13.22; 5.80)	1.000	0.10 (−0.03; 0.23)	0.206	−0.25 (−2.83, 2.33)	1.000	143.80 (−123.62; 411.22)	0.594
**48 hours**								
CWI vs CON	4.55 (−4.96; 14.06)	0.756	0.02 (−0.11; 0.15)	1.000	−1.09 (−3.67; 1.49)	0.937	55.90 (−211.52; 323.32)	1.000
HWI vs CON	−0.35 (−9.86; 9.16)	1.000	0.11 (−0.02; 0.24)	0.135	−0.21 (−2.79; 2.37)	1.000	58.40 (−209.02; 325.82)	1.000
HWI vs CWI	−4.90 (−14.41; 4.61)	0.652	0.09 (−0.04; 0.22)	0.304	0.88 (−1.70; 3.46)	1.000	2.50 (−254.92; 269.92)	1.000
**72 hours**								
CWI vs CON	2.00 (−7.51; 11.51)	1.000	0.02 (−0.11; −0.15)	1.000	−0.36 (−2.94; 2.22)	1.000	23.30 (−244.12; 290.72)	1.000
HWI vs CON	0.57 (−8.94; 10.08)	1.000	0.14 (0.01; 0.27)	**0.032**	1.37 (−1.21; 3.95)	0.612	28.40 (−239.02; 295.82)	1.000
HWI vs CWI	−1.43 (−10.94; 8.08)	1.000	0.12 (−0.01; 0.25)	0.087	1.73 (−0.85; 4.31)	0.326	5.10 (−262.32; 272.52)	1.000

* Baseline time point as a reference; CWI = cold water immersion group, HWI = hot water immersion group, CON = control group. P-values adjusted for multiple comparisons with Bonferroni method.

#### Muscle swelling.

Significant main effects of time (p < 0.001) and intervention (p = 0.046) but no time*intervention interaction (p = 0.057) were found. Muscle swelling was significantly greater in the HWI group compared to CON at 24 h and 72 h follow-up while there was no difference between the CWI group and the other groups (HWI and CON) throughout 72 h follow-up (Table 3, Fig 4B).

#### Delayed onset of muscle soreness.

Analysis of DOMS revealed a significant main effect of time (p < 0.001) but no intervention effect (p = 0.752) or time*intervention interaction (p = 0.454). Even though DOMS tended to decline faster in the CWI group, no significant between-group differences were evident at any time point (Table 3, Fig 4C).

#### Creatine kinase.

Baseline CK values showed high individual variability (CWI: 111.0 ± 41.5U/l, HWI: 214.3 ± 180.9U/l, CON: 119.8 ± 50.2U/l). A significant main effect of time (p < 0.001) was observed, but there was no intervention effect (p = 0.696) or time*intervention interaction (p = 0.280). CK was significantly higher in the HWI group compared to CON at 24 h. No other significant between-group differences in CK levels were detected throughout 72 h follow-up (Table 3, Fig 4D).

## Discussion

This study aimed to compare the effects of post-exercise CWI and HWI on acute physiological responses and recovery in healthy recreationally active women. The findings of this study demonstrate no difference between post-exercise CWI and HWI regarding subjective and objective recovery. Despite distinct acute physiological responses to CWI and HWI, neither intervention improved subjective or objective recovery outcomes during a 72 h follow-up period.

The acute response of SmO_2_ showed no between-group differences immediately following water immersion. However, a subsequent decrease in the CWI group resulted in significantly lower values compared to the HWI group at 20 min and to both the HWI group and CON at the 30 min follow-up. SmO_2_ increased in both the HWI and CWI groups following water immersion, peaking immediately after immersion (Fig 3A). Previous research [41–43] highlights that water temperature and immersion duration primarily influence acute physiological response by affecting tissue and core temperature [10] and subsequent tissue perfusion. The thermal stress applied during HWI, known to cause peripheral vasodilation [41], combined with the exercise-induced increase in muscle perfusion and temperature, likely explains the immediate peak in SmO_2_ following HWI. In contrast, the rise in SmO_2_ after CWI seems unexpected, as cooling typically induces vasoconstriction and reduces perfusion via the central pooling of blood [44]. However, the observed increase in oxygen supply in our study may be attributed, at least in part, to neurogenic activation leading to the release of dilating substances, such as nitric oxide, in the endothelium of the blood vessels [45]. This so-called “cold-induced vasodilation” is proposed to occur after a few minutes of cold exposure to protect against cold-induced injuries, such as frostbite [46]. The study by Roberts et al. supports this explanation, reporting elevated SmO_2_ during post-exercise CWI due to increased blood volume from vasodilation [11]. Alternatively, Ihsan et al. proposed that reduced muscle metabolic activity during cooling could account for increased SmO_2_ despite a reduced muscle blood flow [47]. These contradictory findings may arise from different haemodynamic responses induced by various exercise modalities (e.g., endurance vs resistance exercise [48]) and disparities in the immersion protocols. As we did not measure SmO_2_ or blood flow during the immersion period, we can only hypothesise that cold-induced vasodilation, rather than reduced metabolic activity, explains the rise in SmO_2_ immediately after CWI, given the similarity of our protocol to that of Roberts et al. [11].

After the initial peak, CWI led to a gradual decrease in SmO_2_, resulting in significantly lower values compared to the HWI group at 20 and 30 min, and compared to CON at 30 min. The decline in SmO_2_ of 3.9%, 11.5%, and 15.7% at 10, 20, and 30 min following CWI aligns with previous studies showing reduced SmO_2_ compared to CON following post-exercise CWI [11,49]. This biphasic response, where vasodilation persists for up to 10 minutes before transitioning to vasoconstriction [42], may partially underlie the observed drop in SmO_2_, reflecting short-term microvascular adaptations to cold exposure.

Following immersion, T_core_ initially showed a similar decline in the CWI group and CON. In contrast, HWI maintained T_core_ at an elevated level, resulting in significantly higher temperatures than in the CWI group immediately after immersion (postInt) and at 30 min (Fig 3B). While a higher post-immersion T_core_ was expected following HWI [9,43], our expectation of a more substantial and faster decrease in T_core_ after CWI, as reported in previous studies [50,51], was not met. Interestingly, this aligns with findings from Menzies and colleagues, who reported that the decline in T_core_ in the CWI group was not significantly different from CON up to 15 min post-immersion [52]. They suggested that protocol-specific factors, such as immersion depth and duration, may influence post-immersion temperature stabilisation. Although Menzies et al. did not measure SmO_2_, they reported a significant decrease in superficial femoral blood flow following CWI. These results support our hypothesis that the applied CWI protocol may have induced significant changes in post-immersion peripheral blood flow, leading to a centralisation of blood, which could have mitigated a more pronounced drop in T_core_ compared to CON [9]. Additionally, the insulating effect of subcutaneous adipose tissue, which has been shown to directly influence the magnitude of T_core_ changes after both CWI and HWI [53], could explain the different T_core_ kinetics observed in our female study population. Indeed, the aforementioned studies [50,51] employed a milder but longer CWI protocol (15 and 14 min at 15°C) on male participants, differing from our protocol (10 min at 10 ± 0.5°C) and our female cohort.

The substantial impact of CWI was also observed in the acute reduction of T_skin_. Following CWI, T_skin_ decreased by 48.4% (to 15.2°C) and remained significantly lower than in the HWI group and CON throughout the 30 min follow-up. This finding aligns with the results of Hohenauer et al. [24], who used an identical CWI protocol and Stephens et al., who reported post-exercise T_skin_ reductions of 6–18°C following CWI [54]. In contrast, Roberts et al. reported a less pronounced but more sustained reduction in T_skin_, with a minimum of ~25°C, using the same CWI protocol [11]. Notably, the study of Roberts et al. involved healthy, active men. Consistent with these observations, Klimek et al. reported a more sustained decrease in T_skin_ in men than in women following whole-body cryotherapy, supporting the idea of gender difference in skin cooling and rewarming [55].

Although CWI and HWI induced significantly different acute physiological responses, no differences in either objective or subjective recovery parameters were observed between the two immersion groups during 72 h follow-up. Our findings, which showed no significant differences in recovery of MVIC between the CWI, HWI, and CON groups, are consistent with recent reviews that focused on physically active men [7,8,15]. At 24 h, MVIC decreased by 38.2 ± 17.7%, 37.0 ± 14.5%, and 43.4 ±12.9% in CWI, HWI, and CON groups, respectively. These reductions suggest a large impairment in muscle function, which aligns with findings by Miyama and Nosaka [56], who employed the same exercise protocol in non-resistance-experienced men. Interestingly, only a 20% reduction in MVIC was reported following the identical exercise protocol in active women [24] and only a 30% reduction in active men [23]. Additionally, Hohenauer et al. observed significantly lower DOMS in the CWI group compared to CON during the 72 h follow-up [24]. This contrasts with our results, where no differences in DOMS were found between any of the groups despite using the same protocol and study population. Prior research has shown that experience with eccentric exercise protects against functional loss and DOMS in subsequent exercise sessions [57]. The inconsistent results from the reported studies suggest that responses to eccentric exercise can be highly individual, regardless of sex. The female participants in the present study may have been less experienced in eccentric exercise than more trained or athletic individuals, which could explain the greater functional losses observed following the drop-jump protocol. Given the substantial MVIC losses observed in the present study, it is plausible that the water immersion protocols were not effective enough to significantly enhance recovery by reducing DOMS. While CWI has been moderately studied, yielding mixed results on its efficacy in promoting recovery during 2-–72 h follow-up, comprehensive data on using post-exercise heat applications or HWI for recovery are lacking. However, a recent study by Sautillet et al. showed that a tailored HWI protocol (41°C for ~25 min) successfully mitigates the decline in late-phase rate of force development and, therefore, was more efficient compared to CWI in restoring neuromuscular function and recovery of DOMS in a male population [58]. This again suggests that the temperature and duration of immersion play a critical role in recovery outcomes. Though evidence supporting a clear effect of post-exercise CWI on recovery is limited, some studies have suggested its potential benefits in reducing DOMS [18,24,28]. However, the study by Broatch et al. demonstrated that the perceived benefits of CWI (e.g., DOMS) may be largely placebo-driven [59]. Their findings showed that participants in a thermoneutral placebo condition experienced recovery outcomes similar to those in the CWI group, highlighting the role of belief and expectation in recovery. These findings, together with other recent studies, suggest that the beneficial effects of CWI might be partially explained by psychological factors rather than solely on physiological mechanisms [59–61].

While we did not observe appreciable differences in CK levels and muscle swelling between the CWI and HWI group or CON, HWI resulted in significantly greater muscle swelling at 24 h and increased CK levels at 24 h and 72 h compared to CON. The use of cold is generally proposed to reduce the acute inflammatory response and oedema formation by lowering tissue temperature and blood circulation [62]. Additionally, cold-induced analgesia is attributed to decreased tissue temperature, which reduces the affected area’s metabolic rate and oxygen demand [63]. However, in line with recently published reviews [7,8], we did not observe a beneficial effect of CWI on CK levels or muscle swelling. We thus failed to establish a medium-term effect of cryotherapy on inflammation. CK is a commonly used marker in the assessment of muscle-damaging exercise for inflammatory responses. In our study, CK levels increased by an average of 469% at 24 h compared to BL, with considerable variability among individual subjects. Given that other factors, such as age, sex, and ethnicity, may also affect serum CK levels, the validity of CK as a sole indicator of exercise-induced muscle damage has been questioned in the literature [40]. One of the few studies comparing CWI and HWI to a control condition found reduced CK levels and swelling at 24 h post-exercise in the CWI group (14 min at 15°C) compared to CON and reduced CK levels in the HWI group (14 min at 38°C) compared to CON at 48 h post-exercise [18]. While their study reported reductions in CK levels at specific time points for both the CWI and HWI groups, highly variable responses were observed within the control groups. Our experiment observed no differences in muscle swelling between the CWI and HWI group or CON. However, significantly greater swelling was found in the HWI group compared to CON at specific time points. These findings suggest that muscle swelling is not solely dependent on the hydrostatic pressure but on different factors during water immersion, such as shear responses in the superficial femoral artery and blood flow, respectively [64]. Based on the findings in the current study, drawing definitive conclusions about the inflammatory response to cold or hot water immersion following a 5x20 muscle-damaging drop-jump exercise protocol remains challenging.

This study is not without limitations. We did not obtain information from participants regarding their preferences, beliefs, or prior experiences with specific immersion temperatures. Psychological, social, and contextual factors have been shown to play a major role in functional recovery [65], suggesting that neither CWI nor HWI can be recommended as a recovery modality without considering individual preferences. Additionally, the assessment of sleep quality would have been another interesting variable to evaluate, as it is known to affect recovery [66]. However, as our exercise intervention was carried out during the day, we assume that none of the water immersion protocols affected sleep quality. Furthermore, the selected standardised exercise protocol is not reflective of a functional training or competition scenario and therefore, the findings cannot be directly extrapolated into a real-world setting. Another limitation concerns the hormonal status of participants, as oestrogen is thought to have a protective effect on exercise-induced muscle damage [67]. Due to limited resources, we were not able to align measurements with the menstrual cycles of female participants. However, 63% of our study participants were using hormonal contraceptives, and the different cycle phases were equally represented across all groups (Table 1). Further research in this area is needed, incorporating different water immersion protocols in functional and athletic exercises within a female population. Future studies should also investigate the mechanisms behind the observed discrepancy between acute physiological changes and recovery outcomes. Incorporating measurement of tissue temperature, and personal preferences into future research could provide more comprehensive evidence to guide recovery practices for female athletes.

## Conclusion

In conclusion, this study found that, in female participants, CWI applied after a standardised drop-jump exercise protocol significantly reduces SmO_2_ and T_skin_. In contrast, HWI led to a significant increase in T_core_ and T_skin_. However, despite these distinct acute physiological effects, no measurable differences in recovery parameters were observed over the following 24–72 hours. Neither CWI nor HWI improved subjective or objective recovery characteristics compared to passive CON. These findings highlight the complex relationship between acute physiological responses and long-term recovery outcomes in women. They also underscore the importance of further investigating female participants in recovery research, as most previous studies have focused on male participants. For female athletes, our results suggest that the choice between CWI and HWI does not significantly affect long-term recovery.

## Supporting information

S1 File CONSORT checklist. (PDF)

S2 File Research protocol. (PDF)

S1 Table Mean values (±SD) of physiological parameters at each time point for each intervention. (DOCX)

S2 Table Mean values (±SD) of recovery parameters at each time point for each intervention. (DOCX)

S3 Table Effect sizes (Hedge’s G) of physiological parameters with lower and upper limits. (DOCX)

S4 Table Effect sizes (Hedge’s G) of recovery parameters with lower and upper limits. (DOCX)

S3 Dataset Raw data used for statistical analysis. (XLSX)

## References

[1] Hyldahl RD, Hubal MJ. Lengthening our perspective: morphological, cellular, and molecular responses to eccentric exercise. Muscle Nerve. 2014;49(2):155–70. doi: 10.1002/mus.24077 . 24030935

[2] Clarkson PM, Hubal MJ. Exercise-induced muscle damage in humans. Am J Phys Med Rehabil. 2002;81(11 Suppl):S52-69. doi: 10.1097/00002060-200211001-00007 12409811

[3] Clarkson PM, Nosaka K, Braun B. Muscle function after exercise-induced muscle damage and rapid adaptation. Med Sci Sports Exerc. 1992;24(5):512–20. doi: 10.1249/00005768-199205000-00004 1569847

[4] Järvinen TA, Järvinen TL, Kääriäinen M, Kalimo H, Järvinen M. Muscle injuries: biology and treatment. Am J Sports Med. 2005;33(5):745–64. doi: 10.1177/0363546505274714 15851777

[5] Reilly T, Ekblom B. The use of recovery methods post-exercise. J Sports Sci. 2005;23(6):619–27. doi: 10.1080/02640410400021302 16195010

[6] Barnett A. Using recovery modalities between training sessions in elite athletes: does it help? Sports Med. 2006;36(9):781–96. doi: 10.2165/00007256-200636090-00005 . 16937953

[7] Moore E, Fuller JT, Bellenger CR, Saunders S, Halson SL, Broatch JR, et al. Effects of cold-water immersion compared with other recovery modalities on athletic performance following acute strenuous exercise in physically active participants: a systematic review, meta-analysis, and meta-regression. Sports Med. 2023;53(3):687–705. doi: 10.1007/s40279-022-01800-1 36527593

[8] Moore E, Fuller JT, Buckley JD, Saunders S, Halson SL, Broatch JR, et al. Impact of cold-water immersion compared with passive recovery following a single bout of strenuous exercise on athletic performance in physically active participants: a systematic review with meta-analysis and meta-regression. Sports Med. 2022;52(7):1667–88. doi: 10.1007/s40279-022-01644-9 35157264 PMC9213381

[9] Wilcock IM, Cronin JB, Hing WA. Physiological response to water immersion: a method for sport recovery? Sports Med. 2006;36(9):747–65. doi: 10.2165/00007256-200636090-00003 . 16937951

[10] White GE, Wells GD. Cold-water immersion and other forms of cryotherapy: physiological changes potentially affecting recovery from high-intensity exercise. Extrem Physiol Med. 2013;2(1):26. doi: 10.1186/2046-7648-2-26 24004719 PMC3766664

[11] Roberts LA, Muthalib M, Stanley J, Lichtwark G, Nosaka K, Coombes JS, et al. Effects of cold water immersion and active recovery on hemodynamics and recovery of muscle strength following resistance exercise. Am J Physiol Regul Integr Comp Physiol. 2015;309(4):R389-98. doi: 10.1152/ajpregu.00151.2015 26062633

[12] Hohenauer E, Costello JT, Stoop R, Küng UM, Clarys P, Deliens T, et al. Cold-water or partial-body cryotherapy? Comparison of physiological responses and recovery following muscle damage. Scand J Med Sci Sports. 2018;28(3):1252–62. DOI: doi: 10.1111/sms.13014 29130570

[13] Eston R, Peters D. Effects of cold water immersion on the symptoms of exercise-induced muscle damage. J Sports Sci. 1999;17(3):231–8. doi: 10.1080/026404199366136 . 10362390

[14] Vaile J, O’Hagan C, Stefanovic B, Walker M, Gill N, Askew CD. Effect of cold water immersion on repeated cycling performance and limb blood flow. Br J Sports Med. 2011;45(10):825–9. doi: 10.1136/bjsm.2009.067272 20233843

[15] Chaillou T, Treigyte V, Mosely S, Brazaitis M, Venckunas T, Cheng AJ. Functional impact of post-exercise cooling and heating on recovery and training adaptations: application to resistance, endurance, and sprint exercise. Sports Med Open. 2022;8(1):37. doi: 10.1186/s40798-022-00428-9 35254558 PMC8901468

[16] Ihsan M, Abbiss CR, Allan R. Adaptations to post-exercise cold water immersion: friend, foe, or futile? Front Sports Act Living. 2021;3:714148. doi: 10.3389/fspor.2021.714148 34337408 PMC8322530

[17] Jackman JS, Bell PG, Van Someren K, Gondek MB, Hills FA, Wilson LJ, et al. Effect of hot water immersion on acute physiological responses following resistance exercise. Front Physiol. 2023;14:1213733. doi: 10.3389/fphys.2023.1213733 37476688 PMC10354234

[18] Vaile J, Halson S, Gill N, Dawson B. Effect of hydrotherapy on the signs and symptoms of delayed onset muscle soreness. Eur J Appl Physiol. 2008;102(4):447–55. doi: 10.1007/s00421-007-0605-6 17978833

[19] Skurvydas A, Kamandulis S, Stanislovaitis A, Streckis V, Mamkus G, Drazdauskas A. Leg immersion in warm water, stretch-shortening exercise, and exercise-induced muscle damage. J Athl Train. 2008;43(6):592–9. doi: 10.4085/1062-6050-43.6.592 19030137 PMC2582551

[20] Sautillet B, Bourdillon N, Millet GP, Lemaître F, Cozette M, Delanaud S, et al. Hot water immersion: maintaining core body temperature above 38.5°C mitigates muscle fatigue. Scand J Med Sci Sports. 2024;34(1):e14503. doi: 10.1111/sms.14503 37747708

[21] Cowan SM, Kemp JL, Ardern CL, Thornton JS, Rio EK, Bruder AM, et al. Sport and exercise medicine/physiotherapy publishing has a gender/sex equity problem: we need action now! Br J Sports Med. 2023;57(7):401–7. doi: 10.1136/bjsports-2022-106055 36631242

[22] Karastergiou K, Smith SR, Greenberg AS, Fried SK. Sex differences in human adipose tissues - the biology of pear shape. Biol Sex Differ. 2012;3(1):13. doi: 10.1186/2042-6410-3-13 22651247 PMC3411490

[23] Ferreira-Junior JB, Bottaro M, Vieira A, Siqueira AF, Vieira CA, Durigan JL, et al. One session of partial-body cryotherapy (-110°C) improves muscle damage recovery. Scand J Med Sci Sports. 2015;25(5):e524-30. doi: 10.1111/sms.12353 25556301

[24] Hohenauer E, Costello JT, Deliens T, Clarys P, Stoop R, Clijsen R. Partial-body cryotherapy (-135°C) and cold-water immersion (10°C) after muscle damage in females. Scand J Med Sci Sports. 2020;30(3):485–95. doi: 10.1111/sms.13593 31677292 PMC7027844

[25] Nuttall FQ. Body mass index: obesity, BMI, and health: a critical review. Nutr Today. 2015;50(3):117–28. doi: 10.1097/nt.0000000000000092 . 27340299 PMC4890841

[26] Katch V, McArdle W, Katch F. Energy expenditure during rest and physical activity. McArdle WD, Katch FI, Katch VL eds. Essentials of exercise physiology. 4th ed. Baltimore, MD: Lippincott Williams & Wilkins; 2011, 237–62.

[27] Howatson G, Goodall S, van Someren KA. The influence of cold water immersions on adaptation following a single bout of damaging exercise. Eur J Appl Physiol. 2009;105(4):615–21. doi: 10.1007/s00421-008-0941-1 19034491

[28] Hohenauer E, Taeymans J, Baeyens J-P, Clarys P, Clijsen R. The effect of post-exercise cryotherapy on recovery characteristics: a systematic review and meta-analysis. PLoS One. 2015;10(9):e0139028. doi: 10.1371/journal.pone.0139028 26413718 PMC4586380

[29] McIntyre RD, Zurawlew MJ, Oliver SJ, Cox AT, Mee JA, Walsh NP. A comparison of heat acclimation by post-exercise hot water immersion and exercise in the heat. J Sci Med Sport. 2021;24(8):729–34. doi: 10.1016/j.jsams.2021.05.008 34116919

[30] Zurawlew MJ, Walsh NP, Fortes MB, Potter C. Post-exercise hot water immersion induces heat acclimation and improves endurance exercise performance in the heat. Scand J Med Sci Sports. 2016;26(7):745–54. doi: 10.1111/sms.12638 26661992

[31] Selfe J, Alexander J, Costello JT, May K, Garratt N, Atkins S, et al. The effect of three different (-135°C) whole body cryotherapy exposure durations on elite rugby league players. PLoS One. 2014;9(1):e86420. doi: 10.1371/journal.pone.0086420 24489726 PMC3906033

[32] Ryan TE, Southern WM, Reynolds MA, McCully KK. A cross-validation of near-infrared spectroscopy measurements of skeletal muscle oxidative capacity with phosphorus magnetic resonance spectroscopy. J Appl Physiol (1985). 2013;115(12):1757–66. doi: 10.1152/japplphysiol.00835.2013 24136110 PMC3882936

[33] Travers GJ, Nichols DS, Farooq A, Racinais S, Périard JD. Validation of an ingestible temperature data logging and telemetry system during exercise in the heat. Temperature (Austin). 2016;3(2):208–19. doi: 10.1080/23328940.2016.1171281 27857951 PMC4965001

[34] Bongers C, Daanen HAM, Bogerd CP, Hopman MTE, Eijsvogels TMH. Validity, reliability, and inertia of four different temperature capsule systems. Med Sci Sports Exerc. 2018;50(1):169–75. doi: 10.1249/mss.0000000000001403 28816921

[35] Bogerd CP, Velt KB, Annaheim S, Bongers CCWG, Eijsvogels TMH, Daanen HAM. Comparison of two telemetric intestinal temperature devices with rectal temperature during exercise. Physiol Meas. 2018;39(3):03NT01. doi: 10.1088/1361-6579/aaad52 29406308

[36] Hohenauer E, Clarys P, Baeyens J-P, Clijsen R. Non-invasive assessments of subjective and objective recovery characteristics following an exhaustive jump protocol. J Vis Exp. 2017;(124):55612. doi: 10.3791/55612 28654037 PMC5608348

[37] Warren GL, Lowe DA, Armstrong RB. Measurement tools used in the study of eccentric contraction-induced injury. Sports Med. 1999;27(1):43–59. doi: 10.2165/00007256-199927010-00004 10028132

[38] Chilibeck PD, Stride D, Farthing JP, Burke DG. Effect of creatine ingestion after exercise on muscle thickness in males and females. Med Sci Sports Exerc. 2004;36(10):1781–8. doi: 10.1249/01.mss.0000142301.70419.c6 15595301

[39] Nijholt W, Scafoglieri A, Jager-Wittenaar H, Hobbelen JSM, van der Schans CP. The reliability and validity of ultrasound to quantify muscles in older adults: a systematic review. J Cachexia Sarcopenia Muscle. 2017;8(5):702–12. doi: 10.1002/jcsm.12210 . 28703496 PMC5659048

[40] Baird MF, Graham SM, Baker JS, Bickerstaff GF. Creatine-kinase- and exercise-related muscle damage implications for muscle performance and recovery. J Nutr Metab. 2012;2012:960363. doi: 10.1155/2012/960363 22288008 PMC3263635

[41] Bonde-Petersen F, Schultz-Pedersen L, Dragsted N. Peripheral and central blood flow in man during cold, thermoneutral, and hot water immersion. Aviat Space Environ Med. 1992;63(5):346–50. 1599379

[42] Gregson W, Black MA, Jones H, Milson J, Morton J, Dawson B, et al. Influence of cold water immersion on limb and cutaneous blood flow at rest. Am J Sports Med. 2011;39(6):1316–23. doi: 10.1177/0363546510395497 . 21335348

[43] Weston CF, O’Hare JP, Evans JM, Corrall RJ. Haemodynamic changes in man during immersion in water at different temperatures. Clin Sci (Lond). 1987;73(6):613–6. doi: 10.1042/cs0730613 3319357

[44] Tipton MJ. The initial responses to cold-water immersion in man. Clin Sci (Lond). 1989;77(6):581–8. doi: 10.1042/cs0770581 2691172

[45] Daanen HA. Finger cold-induced vasodilation: a review. Eur J Appl Physiol. 2003;89(5):411–26. doi: 10.1007/s00421-003-0818-2 12712346

[46] Yasukochi Y, Sera T, Kohno T, Nakashima Y, Uesugi M, Kudo S. Cold-induced vasodilation response in a Japanese cohort: insights from cold-water immersion and genome-wide association studies. J Physiol Anthropol. 2023;42(1):2. doi: 10.1186/s40101-023-00319-2 36890596 PMC9993636

[47] Ihsan M, Watson G, Lipski M, Abbiss CR. Influence of postexercise cooling on muscle oxygenation and blood volume changes. Med Sci Sports Exerc. 2013;45(5):876–82. doi: 10.1249/MSS.0b013e31827e13a2 23247707

[48] Heffernan KS, Kelly EE, Collier SR, Fernhall B. Cardiac autonomic modulation during recovery from acute endurance versus resistance exercise. Eur J Cardiovasc Prev Rehabil. 2006;13(1):80–6. doi: 10.1097/01.hjr.0000197470.74070.46 . 16449868

[49] Stanley J, Peake JM, Coombes JS, Buchheit M. Central and peripheral adjustments during high-intensity exercise following cold water immersion. Eur J Appl Physiol. 2014;114(1):147–63. doi: 10.1007/s00421-013-2755-z 24158407

[50] De Paula F, Escobar K, Ottone V, Aguiar P, Aguiar de Matos M, Duarte T, et al. Post-exercise cold-water immersion improves the performance in a subsequent 5-km running trial. Temperature (Austin). 2018;5(4):359–70. doi: 10.1080/23328940.2018.1495023 30574528 PMC6298493

[51] Vaile J, Halson S, Gill N, Dawson B. Effect of hydrotherapy on recovery from fatigue. Int J Sports Med. 2008;29(7):539–44. doi: 10.1055/s-2007-989267 18058595

[52] Menzies C, Clarke ND, Pugh CJA, Steward CJ, Thake CD, Cullen T. Post-exercise hot or cold water immersion does not alter perception of effort or neuroendocrine responses during subsequent moderate-intensity exercise. Exp Physiol. 2024;109(9):1505–16. doi: 10.1113/ep091932 38970776 PMC11363106

[53] Stephens JM, Halson SL, Miller J, Slater GJ, Chapman DW, Askew CD. Effect of body composition on physiological responses to cold-water immersion and the recovery of exercise performance. Int J Sports Physiol Perform. 2018;13(3):382–9. doi: 10.1123/ijspp.2017-0083 28787237

[54] Stephens JM, Halson S, Miller J, Slater GJ, Askew CD. Cold-water immersion for athletic recovery: one size does not fit all. Int J Sports Physiol Perform. 2017;12(1):2–9. doi: 10.1123/ijspp.2016-0095 . 27294485

[55] Klimek AT, Lubkowska A, Szyguła Z, Frączek B, Chudecka M. The influence of single whole body cryostimulation treatment on the dynamics and the level of maximal anaerobic power. Int J Occup Med Environ Health. 2011;24(2):184–91. doi: 10.2478/s13382-011-0017-z 21590430

[56] Miyama M, Nosaka K. Muscle damage and soreness following repeated bouts of consecutive drop jumps. Adv Exerc Sports Physiol. 2004;10(3):63–9.

[57] Nosaka K, Sakamoto K, Newton M, Sacco P. How long does the protective effect on eccentric exercise-induced muscle damage last? Med Sci Sports Exerc. 2001;33(9):1490–5. doi: 10.1097/00005768-200109000-00011 . 11528337

[58] Sautillet B, Bourdillon N, Millet GP, Billaut F, Hassar A, Moufti H, et al. Hot but not cold water immersion mitigates the decline in rate of force development following exercise-induced muscle damage. Med Sci Sports Exerc. 2024;56(12):2362–71. doi: 10.1249/mss.0000000000003513 38967392

[59] Broatch JR, Petersen A, Bishop DJ. Postexercise cold water immersion benefits are not greater than the placebo effect. Med Sci Sports Exerc. 2014;46(11):2139–47. doi: 10.1249/mss.0000000000000348 24674975

[60] Versey NG, Halson SL, Dawson BT. Water immersion recovery for athletes: effect on exercise performance and practical recommendations. Sports Med. 2013;43(11):1101–30. doi: 10.1007/s40279-013-0063-8 23743793

[61] Horgan BG, West NP, Tee N, Drinkwater EJ, Halson SL, Vider J, et al. Acute inflammatory, anthropometric, and perceptual (muscle soreness) effects of postresistance exercise water immersion in junior international and subelite male volleyball athletes. J Strength Cond Res. 2022;36(12):3473–84. doi: 10.1519/jsc.0000000000004122 34537801

[62] Nadler SF, Weingand K, Kruse RJ. The physiologic basis and clinical applications of cryotherapy and thermotherapy for the pain practitioner. Pain Physician. 2004;7(3):395–9. . 16858479

[63] Kwiecien SY, McHugh MP. The cold truth: the role of cryotherapy in the treatment of injury and recovery from exercise. Eur J Appl Physiol. 2021;121(8):2125–42. doi: 10.1007/s00421-021-04683-8 33877402

[64] Thomas KN, van Rij AM, Lucas SJ, Gray AR, Cotter JD. Substantive hemodynamic and thermal strain upon completing lower-limb hot-water immersion; comparisons with treadmill running. Temperature (Austin). 2016;3(2):286–97. doi: 10.1080/23328940.2016.1156215 . 27857958 PMC4964998

[65] Truong LK, Mosewich AD, Holt CJ, Le CY, Miciak M, Whittaker JL. Psychological, social and contextual factors across recovery stages following a sport-related knee injury: a scoping review. Br J Sports Med. 2020;54(19):1149–56. doi: 10.1136/bjsports-2019-101206 32060141 PMC7513260

[66] Charest J, Grandner MA. Sleep and athletic performance: impacts on physical performance, mental performance, injury risk and recovery, and mental health: an update. Sleep Med Clin. 2022;17(2):263–82. doi: 10.1016/j.jsmc.2022.03.006 35659079

[67] Minahan C, Joyce S, Bulmer AC, Cronin N, Sabapathy S. The influence of estradiol on muscle damage and leg strength after intense eccentric exercise. Eur J Appl Physiol. 2015;115(7):1493–500. doi: 10.1007/s00421-015-3133-9 . 25694209

