# Supplementary material for: No acceleration of recovery from exercise-induced muscle damage after cold or hot water immersion in women: A randomised controlled trial
Source: PLoS One. 2025 May 7;20(5):e0322416. doi: 10.1371/journal.pone.0322416 (PMC12057877; doi:10.1371/journal.pone.0322416)
Supplement: S2 File — (PDF) [file pone.0322416.s002.pdf]

Research Protocol

Study Titel: No acceleration of recovery from exercise-induced muscle damage after cold or hot water immersion in women: A randomised controlled trial

Study registration: ClinicalTrials.gov (NCT04902924), Swiss National Clinical Trial Portal (SNCTP000004468)

Study category and rational: Other Clinical Trial of risk category A according to Clin O (Chapter 4) This study is concerned with basic physiological research on the effects of hot and cold water baths on the function of the body and not with the medical application of the devices used. No medication is administered to the test subjects and no tissue samples are taken. Only non-invasive or minimally invasive methods are uses.

Principal Investigator: Dr. Ron Clijsen, University of Applied Sciences and Arts of Southern Switzerland, Weststrasse 8, CH-7302 Landquart, ron.clijsen@supsi.ch, +41 (81) 300 01 75

Monocentric study: RESlab Landquart, University of Applied Sciences and Arts of Southern Switzerland, Weststrasse 8, CH-7302 Landquart.

Background and Rationale

Cold therapies have long been used for treatment after strenuous exercise (Costello et al., 2012). In sports medicine, cold therapy is a common intervention, the effect of which has already been investigated in many studies. Cold therapy applications are used to alleviate the symptoms of subjective muscle soreness and general fatigue (Ascensão et al., 2011; Bailey et al., 2007; Costello et al., 2012; Crystal et al., 2013; Delextrat et al., 2013; Elias et al., 2012; Eston & Peters, 1999; Goodall & Howatson, 2008; Guilhem et al., 2013; Howatson et al., 2009; Ingram et al., 2009; Jakeman et al., 2009; King & Duffield, 2009; Kuligowski et al., 1998; Paddon-Jones & Quigley, 1997; Pointon & Duffield, 2012; Pointon et al., 2011; Pournot et al., 2011; Rowsell et al., 2009; Rupp et al., 2012; Sellwood et al., 2007; Stanley et al., 2013; Tseng et al., 2013). Furthermore, cold therapies are used to positively influence objective parameters such as creatine kinase, lactate, various interleukins or C-reactive proteins (Bastos et al., 2012; Crowe et al., 2007; De Pauw et al., 2014; Guilhem et al., 2013; Heyman et al., 2009; Howatson et al., 2009; King & Duffield, 2009; Leal Junior et al., 2011; Pointon & Duffield, 2012; Tucker et al., 2012; Vaile et al., 2008). However, cold therapy is not only used to optimise subjective and objective recovery parameters but also to improve objective performance (Brade et al., 2014; Duffield et al., 2009; Wegmann et al., 2012)). Cold therapy is described as a procedure that relieves pain and specifically reduces inflammatory reactions following injuries and overuse. The mechanism of action of cooling is said to be the vasoconstrictive effect, which reduces inflammatory reactions by reducing cell metabolism. Banfi and colleagues (Banfi et

al., 2010) recently published results according to which whole-body cooling would not have a negative effect on athletes. However, little evidence was found that favoured whole-body cooling over ‘non-cooling’. In contrast, Leeder and colleagues (Leeder et al., 2012) were able to show that cold water immersion is an effective strategy for alleviating subjective symptoms. These results are consistent with those of Bleakly (Bleakley et al., 2012). In their meta-analysis, Wegmann et al. were able to show that pre-activity cooling can have an effective positive effect on endurance performance (Wegmann et al., 2012).

Hot water immersions have been used in medicine for some time to treat a wide range of conditions. Hydrotherapy has thermal, mechanical and chemical effects and has a vasodilatory and circulation-promoting effect (An et al., 2019). The effect of warm water immersion on recovery after intense physical exertion was first investigated in 1995 (Viitasalo et al., 1995). At that time, it was hypothesised that warm water immersion increased the release of proteins from muscle tissue into the blood and thus promoted the maintenance of neuromuscular performance capacity. Three years later, a research team investigated the effects of warm water immersions in comparison with cold water and contrast therapy and found that cold thermotherapy showed statistically significant differences in the pain data of the test subjects than warm thermotherapy (Kuligowski et al., 1998). However, especially for activities that require excessive force production, they recommended that muscle strength rather than the sensation of pain should be considered as the decisive factor when returning to training or competition. More recent studies comparing hot water therapy with cold water therapy found that while cold water therapy is still more effective, hot water therapy also showed effects on various objective recovery parameters and tests measured after intense exercise (Ascensão et al., 2011; Vaile et al., 2008). Different protocols, for example in relation to water temperature, duration and immersion depth, as well as a lack of answers regarding the effect of warm water immersion on recovery, still do not allow any clear conclusions to be drawn (Versey et al., 2013).

At present, there is a limited number of studies in the literature that have examined the comparative effects of cold and warm water immersion on recovery.

Study goals and objective(s)

The aim of this study is to investigate the effectiveness of repeated cold and warm water baths and provide new insights into the appropriate use of this form of application, in healthy females. The study investigates the effect of repeated cold and warm water baths on the maximum voluntary isometric muscle contraction of the thigh muscles, the inflammation parameter from the venous blood, the muscle swelling and the assessment of the subjective feeling of muscle soreness in the legs during 72 hours of recovery. As an exhaustive measure, the test subjects had to complete a total of 5 x 20 maximum vertical drop jumps.

Primary outcomes:

Physiological measurements

[Time of measurement: Baseline, immediately post-exercise, immediately post-intervention, during 30min follow-up (10, 20, 30min)]

- Muscle oxygen saturation (%)
- Heart rate (bpm)
- Skin temperature (°C)
- Core body temperature (°C)

Recovery measurements

[Time of measurement: Baseline, 24h, 48h and 72h post exercise]

- Maximum voluntary isometric contraction (N)
- Muscle swelling (mm)
- Inflammation parameters: creatine kinase (U/L)
- Delayed onset of muscle damage (VAS scale; cm)

Secondary outcomes:

None

Methods

Study design

Randomised, controlled study

Randomisation procedure

Allocation to the different groups (cold water immersion, hot water immersion, control group) occurs randomly (block randomisation) by drawing an envelope.

Blinding procedures

Due to the nature of the study, blinding of participants and assessors to the recovery intervention is not feasible. The responsible statistician is blinded, has no access to the personal data of the test subjects, and is not present when the data is collected.

Eligibility Criteria

Inclusion criteria

- Young healthy women aged 18-35
- No surgical interventions on the musculoskeletal system in the trunk area and on the lower extremities
- Anticonceptives

Exclusion criteria

- Current injuries of any kind affecting the trunk and/or lower extremities
- Injuries to the trunk and/or lower extremities that occurred less than 1 year ago
- Injuries to the trunk and/or lower extremities that occurred more than 1 year ago and are still causing symptoms
- Fear of cold and/or hot water intervention
- smokers
- Taking medication of any kind (including self-purchased medication)
- Pacemakers & cardiac arrhythmias
- Known circulatory problems
- Positive pregnancy test
- Diagnosed skeletal static deviations
- Appendectomy less than 2 years ago
- Raynaud's syndrome

Measurements and Procedures

Recruitment and Screening

It is advertised on the websites (Homepage & Facebook) of the Thim van der Laan University of Applied Science and the University of Applied Sciences and Arts of Southern Switzerland (SUPSI). The exact texts are based on the checklist of the Zurich Cantonal Ethics Committee. During the initial contact, the potential participants are informed about the procedure and risks of the study, its conditions and the amount of compensation. This is based on a checklist. The subject information and consent form are given to the potential participants and they have the opportunity to ask questions. If the volunteer agrees to the information, she will be asked to attend the first contact day. This includes the explanations on site, familiarisation of the experimental set-up, signing the consent form, completion of a health questionnaire and the pregnancy test. The test subjects do not have to expect any costs for this test. A positive urine test leads to exclusion from this study. Only the principal investigator knows the results of the urine tests of the female subjects. Once all tests have been passed, an appointment is made for the intervention. The current regulations of the Canton of Graubünden and those of the FOPH for protection against the coronavirus will be taken into account and implemented when conducting the study.

Muscle-damaging exercise protocol

All participants carry out a muscle-damaging exercise protocol before the allocated recovery intervention. An exercise protocol was chosen to induce muscle damage on the knee extensor muscles. It comprises five sets of 20 drop-jumps from a 0.6m box, with a 2-min break between each set. The study participants will be verbally encouraged, and the correct execution of the protocol will be visually monitored.

Outcome Measures

Physiological outcomes:

All physiological outcomes are measured at baseline, immediately post-exercise, immediately post-intervention, and during 30min follow-up (10, 20, 30min)

Muscle oxygen saturation (SmO_2): The oxygen saturation of the muscles is measured non-invasively using a deep tissue oxygenation monitor (moorVMS-NIRS, moor instruments, www.moor.co.uk). Adhesive electrodes are placed over the muscle for this purpose.

Heart rate (HR): The heart rate is recorded using a heart rate monitor with a chest belt (Polar T31, Polar Inc. Kempele, Finland).

Core temperature (T_{core}): The core body temperature is determined using the e-Celsius® Performance (https://www.bodycap-medical.com). This non-invasive, disposable, ingestible capsule continuously monitors and records core temperature and transmits the data wirelessly to a monitor (BodyCAP medical, Hérouville Saint-Clair, France). The core body temperature is measured before during and after the intervention.

Skin temperature (T_{skin}): The skin temperature of the right thigh is measured using infrared thermal imaging (FLIR A600 series, Emitec Industrial, Rotkreuz Switzerland) and is evaluated using the corresponding data analysis software (FLIR ResearchIR Max). A predefined skin area on the mid-section of the right thigh is manually marked in the software. The mean temperature of the region of interest is used for the analysis.

Recovery outcomes:

All recovery outcomes are measured at baseline, 24, 48, and 72h post-exercise

Measurement of maximum voluntary isometric contraction (MVIC): Muscle strength of the right knee extensor is assessed based on the performance of an MVIC. The MVIC is performed on a custom-designed ergometer chair in 120° knee flexion, 100° hip angle and the right shin fixed to the ergometer with a strap. Muscle strength is measured during maximal knee extension, performed three times for 4s each, and is expressed in Newtons (N). For the analysis, the highest value of the three attempts is used as the MVIC.

Muscle swelling: Muscle swelling is measured using ultrasound imaging (MyLabClassC, Esaote, Genoa, Italy). The images are analysed using the OsiriX DICOM viewer software (OsiriX, Pixmeo SARL, Switzerland).

Perceived muscle soreness (DOMS): The test subjects indicate their subjective feeling of muscle soreness on a visual analogue scale (VAS). The scale will be scaled from ‘0’ (no muscle soreness present) to ‘10’ (greatest imaginable muscle soreness) in cm increments.

Concentration of serum creatine kinase (CK): The measurement of the inflammatory parameter creatine kinase (CK) is carried out via venous blood sampling. The samples are collected in 8.5 ml tubes (BD Vacutainer, Plymouth, UK), centrifuged (Hettich, EBA 20, Baech, Switzerland) and analysed using an automated ultraviolet method (Roche, Basel, Switzerland).

Study Product and Intervention

The Aspen whirlpool is an inflatable pool with four seats and a capacity of 700 liters. The heating of the 168cm x 168cm whirlpool can be adjusted up to 42°C. The water filtration runs automatically. This product is certified and complies with European regulations, directives and standards. The CE marking indicates that the product fulfils the essential requirements of European Directives 2006/95/EC (low voltage), 2004/108/EC (electromagnetic compatibility) 2011/65/EC (restriction of the use of certain substances in appliances) 2009/125/EC (ecodesign of energy-related products).

Recovery interventions
In order to obtain high-quality study results, the test subjects are only exposed to one test condition at a time. There are three test groups.

The following recovery interventions will be carried out:

- Group CWI with repeated cold water immersion
- Group HWI with repeated hot water immersion
- Passive control group (CON)

During all experimental interventions, an investigator is located directly next to the test person for monitoring at all times.

Cold water immersion (CWI): Test subjects in this group are asked to take a cold water bath after carrying out the jump protocol. The application lasts 10 minutes (and is repeated after 2 hours) at a water temperature of 10°C. In the sitting position, the water level must reach up to the sternum.

Hot water immersion (HWI): Test subjects in this group are asked to take a hot water bath after the jump protocol has been carried out. The application lasts 10 minutes (and is repeated after 2 hours) at a water temperature of 40°C. In the sitting position, the water level must reach up to the sternum.

Control Group (CON): The test subjects in the control group are in our laboratory and are asked to lie on their backs for 10 minutes after completing the jumping protocol.

**Test protocol: Time required – maximum 245 min**		
Cold water immersion vs. hot water immersion vs. passive control group		
**Cold water immersion**<br>**1.) Familiarisation (day 1)**<br>Explanation and familiarisation with experimental set-up, review of inclusion and exclusion criteria, written consent, health questionnaire, pregnancy test<br>= 45min	**Hot water immersion**<br>**1.) Familiarisation (day 1)**<br>Explanation and familiarisation with experimental set-up, review of inclusion and exclusion criteria, written consent, health questionnaire, pregnancy test<br>= 45min	**Passive control intervention**<br>**1.) Familiarisation (day 1)**<br>Explanation and familiarisation with experimental set-up, review of inclusion and exclusion criteria, written consent, health questionnaire, pregnancy test<br>= 45min
**2.) Baseline measurements (day 2)**<br>Anthropometric data, menstrual cycle<br>heart rate<br>skin/body core temperature<br>muscle oxygen saturation<br>Inflammation parameter (CK)<br>Rating of delayed onset muscle soreness (VAS)<br>Muscle swelling (ultrasound)<br>Maximal voluntary isometric contraction (3x)<br>= 45min	**2.) Baseline measurements (day 2)**<br>Anthropometric data, menstrual cycle<br>heart rate<br>skin/body core temperature<br>muscle oxygen saturation<br>Inflammation parameter (CK)<br>Rating of delayed onset muscle soreness (VAS)<br>Muscle swelling (ultrasound)<br>Maximal voluntary isometric contraction (3x)<br>= 45min	**2.) Baseline measurements (day 2)**<br>Anthropometric data, menstrual cycle<br>heart rate<br>skin/body core temperature<br>muscle oxygen saturation<br>Inflammation parameter (CK)<br>Rating of delayed onset muscle soreness (VAS)<br>Muscle swelling (ultrasound)<br>Maximal voluntary isometric contraction (3x)<br>= 45min
**3.) Jump log (day 2)**<br>5 x 20 max. vertical drop jumps<br>→ after 30sek:<br>heart rate<br>skin/body core temperature<br>muscle oxygen saturation<br>= 30min	**3.) Jump log (day 2)**<br>5 x 20 max. vertical drop jumps<br>→ after 30sek:<br>heart rate<br>skin/body core temperature<br>muscle oxygen saturation<br>= 30min	**3.) Jump log (day 2)**<br>5 x 20 max. vertical drop jumps<br>→ after 30sek:<br>heart rate<br>skin/body core temperature<br>muscle oxygen saturation<br>= 30min
**4.) Cold water immersion (day 2)**<br>Immersion of 10 min at 10°C<br>= 10min	**4.) Hot water immersion (day 2)**<br>Immersion of 10 min at 40°C<br>= 10min	**4.) Passive supine position (day 2)**<br>10 min rest at room temperature<br>= 10min

**5.-8.) Follow up (day 2)**<br>Directly following intervention (cold water immersion):<br>heart rate<br>skin/body core temperature<br>muscle oxygen saturation<br>Skin/body core temperature<br>= 5min	**5.-8.) Follow up (day 2)**<br>Directly following intervention (hot water immersion):<br>heart rate<br>skin/body core temperature<br>muscle oxygen saturation<br>Skin/body core temperature<br>= 5min	**5.-8.) Follow up (day 2)**<br>Directly following intervention (passive rest):<br>heart rate<br>skin/body core temperature<br>muscle oxygen saturation<br>Skin/body core temperature<br>= 5min
**9.) Second cold water immersion (day 2)**<br>120 min after first immersion: 10 min at 10°C<br>= 10min	**9.) Second hot water immersion (day 2)**<br>120 min after first immersion: 10 min at 40°C<br>= 10min	
**10.-12.) Recovery follow-up (day 3/ 4/ 5)**<br>24, 48, and 72 hours after the intervention:<br><br>Inflammation parameter (CK)<br>Rating of delayed onset muscle soreness (VAS)<br>Muscle swelling (ultrasound)<br>Maximal voluntary isometric contraction (3x)<br><br>= 30min per follow up	**10.-12.) Recovery follow-up (day 3/ 4/ 5)**<br>24, 48, and 72 hours after the intervention:<br><br>Inflammation parameter (CK)<br>Rating of delayed onset muscle soreness (VAS)<br>Muscle swelling (ultrasound)<br>Maximal voluntary isometric contraction (3x)<br><br>= 30min per follow up	**9.-11.) Recovery follow-up (day 3/ 4/ 5)**<br>24, 48, and 72 hours after the intervention:<br><br>Inflammation parameter (CK)<br>Rating of delayed onset muscle soreness (VAS)<br>Muscle swelling (ultrasound)<br>Maximal voluntary isometric contraction (3x)<br><br>= 30min per follow up

Data management and statistical analysis

The power analysis using the G*Power app (Düsseldorf, North Rhine-Westphalia, Germany) showed that a minimum of 24 participants is necessary to ensure a statistical power of 0.80 to detect a parameter difference at the 5% significance level. Considering a possible loss of 20% of the data sets (due to drop-out or incomplete data sets), the minimum sample size is set at 30 participants. The number of subjects in this study is comparable to other studies in the field of thermotherapy (Kuligowski et al., 1998; Pournot et al., 2011; Vaile et al., 2008).

Statistical Analyses

The effectiveness of repeated cold and hot water immersion on recovery and performance will be tested using a mixed-effect model with restricted maximum likelihood method.

Random effect: subjects

Fixed effect: intervention (cold water immersion, hot water immersion, control group), times points (for physiological parameters: baseline, post exercise, post intervention, 10min. 20min, 30min; for recovery parameters: baseline, 24h, 48h, 72h) and their interaction.

The significance level is set at $p < 0.05$, the statistical data analysis is performed with Stata V18 (StataCorp LLC, Texas, USA)

Data safety monitoring committee

All data collected from the study participants will be coded and not passed on to other persons. The personal data and information on the individuals will be stored in paper form in a locked filing cabinet. Only the study supervisor, the head of the research laboratory and the concurrent study director have access, whereby the administration of the documents is the responsibility of the head of the research laboratory.

All digital data is encoded. This means that no conclusions can be drawn about individuals. The digital data is stored and archived on the institute's internal computers and is not disclosed to third parties. Employees who are involved in data processing have no access to personal data and the coding of this data.

Study timeline

Study Duration and Schedule: May 2021 – December 2023

May-December 2021:	Subject information, screening, planning, data collection
Januar-August 2022:	Statistics
September 2022 -Mai 2023:	Writing the article
Juni-Dezember 2023:	Time for publication

Investigator(s)

Investigator
- Ron Clijsen, PhD[1,2,3]
- Hohenauer Erich, PhD[1,2,3]
- Dias Giannina[2]
- Wellauer Vanessa[2]
- Riggi Emilia[4]
- Freitag Livia[2]
- Herten Miriam[2]

[1] University College Physiotherapy Thim van der Laan, Weststrasse 8, 7302 Landquart
[2] University of Applied Sciences and Arts of Southern Switzerland, Rehabilitation and Exercise Science Laboratory RESlab, Rehabilitation and Exercise Science Group, Departement of Business Economics, Health and Social Care, Physiotherapie Graubünden, Weststrasse 8, 7302 Landuart
[3] Vrije Universiteit Brussels, Faculty of Physical Education and Physical Therapy, Pleinlann 2, 1050 Brussels
[4] Department of Business Economics, Health and Social Care, University of Applied Sciences and Arts of Southern Switzerland, Manno, Switzerland

Contact / Study director
Clijsen Ron, PhD
University of Applied Sciences and Arts of Southern Switzerland
Physiotherapy Graubünden
Weststrasse 8, 7302 Landquart
+41 81 300 01 75
ron.clijsen@supsi.ch

Study Centre(s)

University of Applied Sciences and Arts of Southern Switzerland
Physiotherapy Graubünden
Rehabilitation Research Laboratory 2rLab
Rehabilitation and Exercise Science Group
Weststrasse 8
7302 Landquart

GCP Statement

This study will be conducted in compliance with the protocol, the current version of the Declaration of Helsinki, the ICH-GCP or ISO EN 14155 (as far as applicable) as well as all national legal and regulatory requirements.

Funding and Insurance

Funding

The study is funded by the University of Applied Sciences and Arts of Southern Switzerland, Physiotherapy Grisons. The investigators are employed by this institute and are remunerated for their work on this study in accordance with their employment contracts. No further financial support is required.

Insurance

Thim van der Laan AG in Landquart, where the study is being conducted, is covered by public liability insurance with Basler Versicherung.

References

An, J., Lee, I., & Yi, Y. (2019). The Thermal Effects of Water Immersion on Health Outcomes: An Integrative Review. *Int J Environ Res Public Health*, *16*(7). https://doi.org/10.3390/ijerph16071280

Ascensão, A., Leite, M., Rebelo, A. N., Magalhäes, S., & Magalhäes, J. (2011). Effects of cold water immersion on the recovery of physical performance and muscle damage following a one-off soccer match. *J Sports Sci*, *29*(3), 217-225. https://doi.org/10.1080/02640414.2010.526132

Bailey, D. M., Erith, S. J., Griffin, P. J., Dowson, A., Brewer, D. S., Gant, N., & Williams, C. (2007). Influence of cold-water immersion on indices of muscle damage following prolonged intermittent shuttle running. *J Sports Sci*, *25*(11), 1163-1170. https://doi.org/10.1080/02640410600982659

Banfi, G., Lombardi, G., Colombini, A., & Melegati, G. (2010). Whole-body cryotherapy in athletes. *Sports Med*, *40*(6), 509-517. https://doi.org/10.2165/11531940-000000000-00000

Bastos, F. N., Vanderlei, L. C., Nakamura, F. Y., Bertollo, M., Godoy, M. F., Hoshi, R. A., Junior, J. N., & Pastre, C. M. (2012). Effects of cold water immersion and active recovery on post-exercise heart rate variability. *Int J Sports Med*, *33*(11), 873-879. https://doi.org/10.1055/s-0032-1301905

Bleakley, C., McDonough, S., Gardner, E., Baxter, G. D., Hopkins, J. T., & Davison, G. W. (2012). Cold-water immersion (cryotherapy) for preventing and treating muscle soreness after exercise. *Cochrane Database Syst Rev*, *2012*(2), Cd008262. https://doi.org/10.1002/14651858.CD008262.pub2

Brade, C., Dawson, B., & Wallman, K. (2014). Effects of different precooling techniques on repeat sprint ability in team sport athletes. *Eur J Sport Sci*, *14 Suppl 1*, S84-91. https://doi.org/10.1080/17461391.2011.651491

Costello, J. T., Culligan, K., Selfe, J., & Donnelly, A. E. (2012). Muscle, skin and core temperature after -110°c cold air and 8°c water treatment. *PLoS One*, *7*(11), e48190. https://doi.org/10.1371/journal.pone.0048190

Crowe, M. J., O'Connor, D., & Rudd, D. (2007). Cold water recovery reduces anaerobic performance. *Int J Sports Med*, *28*(12), 994-998. https://doi.org/10.1055/s-2007-965118

Crystal, N. J., Townson, D. H., Cook, S. B., & LaRoche, D. P. (2013). Effect of cryotherapy on muscle recovery and inflammation following a bout of damaging exercise. *Eur J Appl Physiol*, *113*(10), 2577-2586. https://doi.org/10.1007/s00421-013-2693-9

De Pauw, K., Roelands, B., Vanparijs, J., & Meeusen, R. (2014). Effect of recovery interventions on cycling performance and pacing strategy in the heat. *Int J Sports Physiol Perform*, *9*(2), 240-248. https://doi.org/10.1123/ijspp.2012-0366

Delextrat, A., Calleja-González, J., Hippocrate, A., & Clarke, N. D. (2013). Effects of sports massage and intermittent cold-water immersion on recovery from matches by basketball players. *J Sports Sci*, *31*(1), 11-19. https://doi.org/10.1080/02640414.2012.719241

Duffield, R., Steinbacher, G., & Fairchild, T. J. (2009). The use of mixed-method, part-body pre-cooling procedures for team-sport athletes training in the heat. *J Strength Cond Res*, *23*(9), 2524-2532. https://doi.org/10.1519/JSC.0b013e3181bf7a4f

Elias, G. P., Varley, M. C., Wyckelsma, V. L., McKenna, M. J., Minahan, C. L., & Aughey, R. J. (2012). Effects of water immersion on posttraining recovery in Australian footballers. *Int J Sports Physiol Perform*, *7*(4), 357-366. https://doi.org/10.1123/ijspp.7.4.357

Eston, R., & Peters, D. (1999). Effects of cold water immersion on the symptoms of exercise-induced muscle damage. *J Sports Sci*, *17*(3), 231-238. https://doi.org/10.1080/026404199366136

Goodall, S., & Howatson, G. (2008). The effects of multiple cold water immersions on indices of muscle damage. *J Sports Sci Med*, *7*(2), 235-241. https://www.ncbi.nlm.nih.gov/pmc/articles/PMC3761456/pdf/jssm-07-235.pdf

Guilhem, G., Hug, F., Couturier, A., Regnault, S., Bournat, L., Filliard, J. R., & Dorel, S. (2013). Effects of air-pulsed cryotherapy on neuromuscular recovery subsequent to exercise-induced muscle damage. *Am J Sports Med*, *41*(8), 1942-1951. https://doi.org/10.1177/0363546513490648

Heyman, E., B, D. E. G., Mertens, I., & Meeusen, R. (2009). Effects of four recovery methods on repeated maximal rock climbing performance. *Med Sci Sports Exerc*, *41*(6), 1303-1310. https://doi.org/10.1249/MSS.0b013e318195107d

Howatson, G., Goodall, S., & van Someren, K. A. (2009). The influence of cold water immersions on adaptation following a single bout of damaging exercise. *Eur J Appl Physiol*, *105*(4), 615-621. https://doi.org/10.1007/s00421-008-0941-1

Ingram, J., Dawson, B., Goodman, C., Wallman, K., & Beilby, J. (2009). Effect of water immersion methods on post-exercise recovery from simulated team sport exercise. *J Sci Med Sport*, *12*(3), 417-421. https://doi.org/10.1016/j.jsams.2007.12.011

Jakeman, J. R., Macrae, R., & Eston, R. (2009). A single 10-min bout of cold-water immersion therapy after strenuous plyometric exercise has no beneficial effect on recovery from the symptoms of exercise-induced muscle damage. *Ergonomics*, *52*(4), 456-460. https://doi.org/10.1080/00140130802707733

King, M., & Duffield, R. (2009). The effects of recovery interventions on consecutive days of intermittent sprint exercise. *J Strength Cond Res*, *23*(6), 1795-1802. https://doi.org/10.1519/JSC.0b013e3181b3f81f

Kuligowski, L. A., Lephart, S. M., Giannantonio, F. P., & Blanc, R. O. (1998). Effect of whirlpool therapy on the signs and symptoms of delayed-onset muscle soreness. *J Athl Train*, *33*(3), 222-228.

https://www.ncbi.nlm.nih.gov/pmc/articles/PMC1320427/pdf/jathtrain00011-0032.pdf

Leal Junior, E. C., de Godoi, V., Mancalossi, J. L., Rossi, R. P., De Marchi, T., Parente, M., Grosselli, D., Generosi, R. A., Basso, M., Frigo, L., Tomazoni, S. S., Bjordal, J. M., & Lopes-Martins, R. A. (2011). Comparison between cold water immersion therapy (CWIT) and light emitting diode therapy (LEDT) in short-term skeletal muscle recovery after high-intensity exercise in athletes--preliminary results. *Lasers Med Sci*, *26*(4), 493-501. https://doi.org/10.1007/s10103-010-0866-x

Leeder, J., Gissane, C., van Someren, K., Gregson, W., & Howatson, G. (2012). Cold water immersion and recovery from strenuous exercise: a meta-analysis. *Br J Sports Med*, *46*(4), 233-240. https://doi.org/10.1136/bjsports-2011-090061

Paddon-Jones, D. J., & Quigley, B. M. (1997). Effect of cryotherapy on muscle soreness and strength following eccentric exercise. *Int J Sports Med*, *18*(8), 588-593. https://doi.org/10.1055/s-2007-972686

Pointon, M., & Duffield, R. (2012). Cold water immersion recovery after simulated collision sport exercise. *Med Sci Sports Exerc*, *44*(2), 206-216. https://doi.org/10.1249/MSS.0b013e31822b0977

Pointon, M., Duffield, R., Cannon, J., & Marino, F. E. (2011). Cold application for neuromuscular recovery following intense lower-body exercise. *Eur J Appl Physiol*, *111*(12), 2977-2986. https://doi.org/10.1007/s00421-011-1924-1

Pournot, H., Bieuzen, F., Duffield, R., Lepretre, P. M., Cozzolino, C., & Hausswirth, C. (2011). Short term effects of various water immersions on recovery from exhaustive intermittent exercise. *Eur J Appl Physiol*, *111*(7), 1287-1295. https://doi.org/10.1007/s00421-010-1754-6

Rowsell, G. J., Coutts, A. J., Reaburn, P., & Hill-Haas, S. (2009). Effects of cold-water immersion on physical performance between successive matches in high-performance junior male soccer players. *J Sports Sci*, *27*(6), 565-573. https://doi.org/10.1080/02640410802603855

Rupp, K. A., Selkow, N. M., Parente, W. R., Ingersoll, C. D., Weltman, A. L., & Saliba, S. A. (2012). The effect of cold water immersion on 48-hour performance testing in collegiate soccer players. *J Strength Cond Res*, *26*(8), 2043-2050. https://doi.org/10.1519/JSC.0b013e318239c3a1

Sellwood, K. L., Brukner, P., Williams, D., Nicol, A., & Hinman, R. (2007). Ice-water immersion and delayed-onset muscle soreness: a randomised controlled trial. *Br J Sports Med*, *41*(6), 392-397. https://doi.org/10.1136/bjsm.2006.033985

Stanley, J., Peake, J. M., & Buchheit, M. (2013). Consecutive days of cold water immersion: effects on cycling performance and heart rate variability. *Eur J Appl Physiol*, *113*(2), 371-384. https://doi.org/10.1007/s00421-012-2445-2

Tseng, C.-Y., Lee, J.-P., Tsai, Y.-S., Lee, S.-D., Kao, C.-L., Liu, T.-C., Lai, C.-H., Harris, M. B., & Kuo, C.-H. (2013). Topical Cooling (Icing) Delays Recovery From Eccentric Exercise–Induced Muscle Damage. *The Journal of Strength & Conditioning Research*, *27*(5). https://journals.lww.com/nsca-jscr/Fulltext/2013/05000/Topical_Cooling__Icing__Delays_Recovery_From.24.aspx

Tucker, T. J., Slivka, D. R., Cuddy, J. S., Hailes, W. S., & Ruby, B. C. (2012). Effect of local cold application on glycogen recovery. *J Sports Med Phys Fitness*, *52*(2), 158-164.

Vaile, J., Halson, S., Gill, N., & Dawson, B. (2008). Effect of hydrotherapy on the signs and symptoms of delayed onset muscle soreness. *Eur J Appl Physiol*, *102*(4), 447-455. https://doi.org/10.1007/s00421-007-0605-6

Versey, N. G., Halson, S. L., & Dawson, B. T. (2013). Water immersion recovery for athletes: effect on exercise performance and practical recommendations. *Sports Med*, *43*(11), 1101-1130. https://doi.org/10.1007/s40279-013-0063-8

Viitasalo, J. T., Niemelä, K., Kaappola, R., Korjus, T., Levola, M., Mononen, H. V., Rusko, H. K., & Takala, T. E. (1995). Warm underwater water-jet massage improves recovery from intense physical exercise. *Eur J Appl Physiol Occup Physiol*, *71*(5), 431-438. https://doi.org/10.1007/bf00635877

Wegmann, M., Faude, O., Poppendieck, W., Hecksteden, A., Fröhlich, M., & Meyer, T. (2012). Pre-cooling and sports performance: a meta-analytical review. *Sports Med*, *42*(7), 545-564. https://doi.org/10.2165/11630550-000000000-00000
