# Supplementary material for: No acceleration of recovery from exercise-induced muscle damage after cold or hot water immersion in women: A randomised controlled trial
Source: PLoS One. 2025 May 7;20(5):e0322416. doi: 10.1371/journal.pone.0322416 (PMC12057877; doi:10.1371/journal.pone.0322416)
Supplement: S1 Table — (DOCX) [file pone.0322416.s003.docx]

**Table S1. Mean values (± SD) of physiological parameters at each time point for each intervention.**

| **Parameters** | **BL** | **postEx** | **postInt** | **10min** | **20min** | **30min** |
| --- | --- | --- | --- | --- | --- | --- |
|  | **Mean (SD)** | **Mean (SD)** | **Mean (SD)** | **Mean (SD)** | **Mean (SD)** | **Mean (SD)** |
| **CON (n=10)** |  |  |  |  |  |  |
| Muscle oxygen saturation [%] | 70.0 (3.7) | 73.4 (5.9) | 74.0 (4.8) | 75.0 (5.4) | 73.5 (6.6) | 73.0 (6.1) |
| Core temperature [°C] | 36.8 (0.4) | 38.3 (0.2) | 37.9 (0.3) | 37.5 (0.2) | 37.3 (0.2) | 37.2 (0.1) |
| Skin temperature [°C] | 30.9 (0.9) | 29.5 (1.7) | 31.8 (1.0) | 32.3 (0.8) | 32.4 (0.8) | 32.5 (0.9) |
| Heart rate [bpm] | 75.0 (15.5) | 189.2 (13.5) | 97.9 (13.7) | 89.5 (11.3) | 85.7 (11.2) | 78.0 (11.9) |
| **CWI (n=10)** |  |  |  |  |  |  |
| Muscle oxygen saturation [%] | 68.7 (4.4) | 70.4 (7.7) | 76.6 (5.8) | 73.6 (5.3) | 67.8 (2.7) | 64.4 (6.4) |
| Core temperature [°C] | 37.1 (0.4) | 38.5 (0.2) | 37.9 (0.2) | 37.6 (0.2) | 37.3 (0.2) | 37.2 (0.2) |
| Skin temperature [°C] | 30.6 (0.9) | 30.0 (0.9) | 15.2 (2.5) | 24.8 (1.2) | 27.9 (1.1) | 29.2 (1.0) |
| Heart rate [bpm] | 72.1 (14.3) | 186.3 (16.9) | 97.9 (12.1) | 77.8 (11.7) | 75.1 (14.5) | 73.2 (14.5) |
| **HWI (n=10)** |  |  |  |  |  |  |
| Muscle oxygen saturation [%] | 68.8 (3.8) | 71.1 (7.1) | 76.6 (3.4) | 75.5 (5.1) | 74.7 (3.2) | 74.4 (3.9) |
| Core temperature [°C] | 37.1 (0.2) | 38.4 (0.3) | 38.5 (0.3) | 37.8 (0.2) | 37.5 (0.2) | 37.5 (0.2) |
| Skin temperature [°C] | 31.2 (1.1) | 29.9 (1.0) | 35.2 (0.7) | 35.3 (0.5) | 34.9 (0.5) | 34.8 (0.7) |
| Heart rate [bpm] | 78.7 (18.8) | 189.5 (9.1) | 103.6 (16.7) | 94.6 (13.9) | 89.1 (12.5) | 88.4 (15.4) |
| CON = control group, CWI = cold water immersion group, HWI = hot water immersion group, BL= baseline time point, postEx= post-exercise, postINT= post intervention, 10min = after 10 min from intervention, 20min = after 20min from intervention and 30min = after 30min from intervention | | | | | | |
