# Supplementary material for: No acceleration of recovery from exercise-induced muscle damage after cold or hot water immersion in women: A randomised controlled trial
Source: PLoS One. 2025 May 7;20(5):e0322416. doi: 10.1371/journal.pone.0322416 (PMC12057877; doi:10.1371/journal.pone.0322416)
Supplement: S2 Table — (DOCX) [file pone.0322416.s004.docx]

**Table S2. Mean values (± SD) of recovery parameters at each time point for each intervention.**

|  | **BL** | **24h** | **48h** | **72h** |
| --- | --- | --- | --- | --- |
|  | **Mean (SD)** | **Mean (SD)** | **Mean (SD)** | **Mean (SD)** |
| **CON (n=10)** |  |  |  |  |
| Maximum voluntary isometric contraction [N] | 52.2 (14.1) | 29.3 (8.7) | 34.7 (12.3) | 40.4 (13.2) |
| Muscle swelling [cm] | 4.6 (0.5) | 4.8 (0.6) | 4.7 (0.6) | 4.6 (0.6) |
| Delayed onset muscle soreness [cm] | 0.1 (0.1) | 6.3 (2.7) | 6.1 (3.1) | 3.3 (2.3) |
| Creatine kinase [U/l] | 119.8 (50.2) | 455.0 (318.2) | 288.3 (200.9) | 191.4 (118.1) |
| **CWI (n=10)** |  |  |  |  |
| Maximum voluntary isometric contraction [N] | 54.8 (14.9) | 33.8 (13.9) | 41.8 (16.4) | 45.0 (17.0) |
| Muscle swelling [cm] | 4.3 (0.5) | 4.6 (0.5) | 4.5 (0.5) | 4.4 (0.5) |
| Delayed onset of muscle soreness [cm] | 0.1 (0.1) | 6.3 (2.6) | 5.0 (2.8) | 2.9 (2.6) |
| Creatine kinase [U/l] | 111.0 (41.5) | 573.5 (488.9) | 335.4 (256.9) | 205.9 (146.2) |
| **HWI (n=10)** |  |  |  |  |
| Maximum voluntary isometric contraction [N] | 65.8 (11.6) | 41.1 (11.4) | 47.9 (10.3) | 54.5 (11.8) |
| Muscle swelling [cm] | 4.8 (0.6) | 5.2 (0.6) | 5.1 (0.5) | 5.0 (0.6) |
| Delayed onset of muscle soreness [cm] | 0.1 (0.1) | 6.1 (2.5) | 5.9 (3.0) | 4.6 (3.1) |
| Creatine kinase [U/l] | 214.3 (180.9) | 820.6 (517.7) | 441.2 (349.2) | 314.3 (321.8) |
| CON = control group, CWI = cold water immersion group, HWI = hot water immersion group, 24h = after 24 hours from intervention, 48h = after 48 hours from intervention and 72h = after 72 hours from intervention | | | | |
