# Supplementary material for: No acceleration of recovery from exercise-induced muscle damage after cold or hot water immersion in women: A randomised controlled trial
Source: PLoS One. 2025 May 7;20(5):e0322416. doi: 10.1371/journal.pone.0322416 (PMC12057877; doi:10.1371/journal.pone.0322416)
Supplement: S3 Table — (DOCX) [file pone.0322416.s005.docx]

**Table S3. Effect sizes (Hedges’ G) of physiological parameters with lower and upper limits.**

| **Comparison** | **Muscle oxygen saturation** | | | **Core temperature** | | | **Skin temperature** | | | **Heart rate** | | |
| --- | --- | --- | --- | --- | --- | --- | --- | --- | --- | --- | --- | --- |
|  | **Effect size** | **Lower** | **Upper** | **Effect size** | **Lower** | **Upper** | **Effect size** | **Lower** | **Upper** | **Effect size** | **Lower** | **Upper** |
| **Baseline** |  |  |  |  |  |  |  |  |  |  |  |  |
| CWI vs CON | -0.28 | -1.39 | 0.83 | -0.87 | -1.87 | 0.13 | 0.33 | -0.80 | 1.45 | 0.35 | -0.78 | 1.47 |
| HWI vs CON | -0.30 | -1.46 | 0.86 | -0.73 | -1.76 | 0.30 | 0.17 | -1.05 | 1.39 | 0.16 | -1.01 | 1.32 |
| HWI vs CWI | -0.02 | -1.14 | 1.09 | 0.14 | -0.84 | 1.12 | -0.16 | -1.34 | 1.02 | -0.19 | -1.31 | 0.92 |
| **postEx** |  |  |  |  |  |  |  |  |  |  |  |  |
| CWI vs CON | -0.67 | -1.79 | 0.44 | -0.26 | -1.25 | 0.74 | 0.72 | -0.41 | 1.85 | 0.35 | -0.78 | 1.47 |
| HWI vs CON | -0.57 | -1.73 | 0.60 | 0.03 | -0.99 | 1.05 | 0.43 | -0.79 | 1.65 | 0.59 | -0.58 | 1.77 |
| HWI vs CWI | 0.11 | -1.00 | 1.22 | 0.29 | -0.69 | 1.26 | -0.29 | -1.47 | 0.89 | 0.25 | -0.87 | 1.36 |
| **postInt** |  |  |  |  |  |  |  |  |  |  |  |  |
| CWI vs CON | 0.63 | -0.48 | 1.75 | 0.37 | -0.63 | 1.36 | -0.59 | -1.71 | 0.54 | -0.03 | -1.15 | 1.10 |
| HWI vs CON | 0.58 | -0.58 | 1.74 | -1.86 | -2.90 | -0.81 | -0.71 | -1.94 | 0.51 | -0.10 | -1.27 | 1.07 |
| HWI vs CWI | -0.06 | -1.17 | 1.05 | -2.22 | -3.23 | -1.21 | -0.13 | -1.30 | 1.05 | -0.08 | -1.19 | 1.04 |
| **10min** |  |  |  |  |  |  |  |  |  |  |  |  |
| CWI vs CON | -0.30 | -1.41 | 0.81 | 0.27 | -0.72 | 1.27 | 0.35 | -0.78 | 1.47 | 1.48 | 0.35 | 2.62 |
| HWI vs CON | 0.09 | -1.07 | 1.25 | -0.61 | -1.64 | 0.42 | -0.22 | -1.44 | 1.00 | -0.02 | -1.19 | 1.14 |
| HWI vs CWI | 0.39 | -0.73 | 1.50 | -0.88 | -1.87 | 0.10 | -0.57 | -1.75 | 0.61 | -1.51 | -2.63 | -0.38 |
| **20min** |  |  |  |  |  |  |  |  |  |  |  |  |
| CWI vs CON | -1.30 | -2.42 | -0.18 | 0.41 | -0.58 | 1.41 | 1.35 | 0.22 | 2.48 | 1.34 | 0.21 | 2.47 |
| HWI vs CON | 0.25 | -0.91 | 1.41 | -0.39 | -1.41 | 0.64 | -0.39 | -1.61 | 0.83 | 0.19 | -0.98 | 1.36 |
| HWI vs CWI | 1.55 | 0.43 | 2.68 | -0.80 | -1.78 | 0.18 | -1.74 | -2.93 | -0.55 | -1.15 | -2.27 | -0.02 |
| **30min** |  |  |  |  |  |  |  |  |  |  |  |  |
| CWI vs CON | -1.98 | -3.11 | -0.85 | 0.81 | -0.19 | 1.81 | 2.03 | 0.88 | 3.17 | 0.59 | -0.53 | 1.72 |
| HWI vs CON | 0.30 | -0.86 | 1.46 | -0.63 | -1.66 | 0.39 | -0.43 | -1.65 | 0.79 | -0.71 | -1.88 | 0.46 |
| HWI vs CWI | 2.28 | 1.14 | 3.42 | -1.45 | -2.44 | -0.46 | -2.46 | -3.67 | -1.25 | -1.30 | -2.42 | -0.18 |
| CON = control group, CWI = cold water immersion group, HWI = hot water immersion group, postEx= post-exercise, postInt= post intervention, 10min = after 10 min from intervention, 20min = after 20min from intervention and 30min = after 30min from intervention | | | | | | | | | | | | |
