# Supplementary material for: No acceleration of recovery from exercise-induced muscle damage after cold or hot water immersion in women: A randomised controlled trial
Source: PLoS One. 2025 May 7;20(5):e0322416. doi: 10.1371/journal.pone.0322416 (PMC12057877; doi:10.1371/journal.pone.0322416)
Supplement: S4 Table — (DOCX) [file pone.0322416.s006.docx]

**Table S3. Effect sizes (Hedges’ G) of recovery parameters with lower and upper limits.**

| **Comparison** | **Maximum voluntary isometric contraction** | | | **Muscle swelling** | | | **Delayed onset of muscle damage** | | | **Creatine kinase** | | |
| --- | --- | --- | --- | --- | --- | --- | --- | --- | --- | --- | --- | --- |
|  | **Effect size** | **Lower** | **Upper** | **Effect size** | **Lower** | **Upper** | **Effect size** | **Lower** | **Upper** | **Effect size** | **Lower** | **Upper** |
| **Baseline** |  |  |  |  |  |  |  |  |  |  |  |  |
| CWI vs CON | -0.11 | -1.31 | 1.09 | 0.01 | -1.30 | 1.32 | -0.03 | -1.35 | 1.29 | -0.10 | -1.34 | 1.13 |
| HWI vs CON | -0.53 | -1.86 | 0.80 | -0.04 | -1.37 | 1.29 | -0.09 | -1.49 | 1.32 | 0.18 | -1.13 | 1.50 |
| HWI vs CWI | -0.42 | -1.66 | 0.82 | -0.05 | -1.32 | 1.22 | -0.06 | -1.38 | 1.26 | 0.29 | -0.98 | 1.56 |
| **24h** |  |  |  |  |  |  |  |  |  |  |  |  |
| CWI vs CON | -0.41 | -1.61 | 0.79 | -0.80 | -2.11 | 0.52 | -0.06 | -1.38 | 1.27 | -0.83 | -2.07 | 0.42 |
| HWI vs CON | -0.24 | -1.57 | 1.09 | -2.00 | -3.35 | -0.64 | 0.04 | -1.37 | 1.44 | -1.35 | -2.68 | -0.02 |
| HWI vs CWI | 0.17 | -1.07 | 1.41 | -1.20 | -2.49 | 0.08 | 0.09 | -1.23 | 1.41 | -0.52 | -1.79 | 0.75 |
| **48h** |  |  |  |  |  |  |  |  |  |  |  |  |
| CWI vs CON | -0.83 | -2.04 | 0.37 | -0.22 | -1.53 | 1.09 | 0.61 | -0.72 | 1.93 | -0.42 | -1.66 | 0.82 |
| HWI vs CON | -0.47 | -1.80 | 0.85 | -1.31 | -2.65 | 0.03 | 0.04 | -1.37 | 1.44 | -0.15 | -1.46 | 1.17 |
| HWI vs CWI | 0.36 | -0.88 | 1.60 | -1.09 | -2.37 | 0.19 | -0.57 | -1.89 | 0.75 | 0.28 | -0.99 | 1.54 |
| **72h** |  |  |  |  |  |  |  |  |  |  |  |  |
| CWI vs CON | -0.43 | -1.63 | 0.77 | -0.22 | -1.53 | 1.09 | 0.18 | -1.14 | 1.50 | -0.24 | -1.47 | 1.00 |
| HWI vs CON | -0.62 | -1.95 | 0.71 | -1.65 | -3.00 | -0.31 | -0.89 | -2.30 | 0.52 | 0.02 | -1.29 | 1.34 |
| HWI vs CWI | -0.19 | -1.43 | 1.05 | -1.43 | -2.72 | -0.14 | -1.07 | -2.40 | 0.26 | 0.26 | -1.01 | 1.53 |
| CON = control group, CWI = cold water immersion group, HWI = hot water immersion group, 24h= after 24 hours from intervention, 48h= after 48 hours from intervention, 72h = after 72 hours from intervention | | | | | | | | | | | | |
